# Global Proteomic Determination of the Poly-Pharmacological Effects of PARP Inhibitors Following Treatment of High-Grade Serous Ovarian Cancer Cells

**DOI:** 10.3390/ijms262411820

**Published:** 2025-12-07

**Authors:** Jesenia M. Perez, Valerie Barrera-Estrada, Carly A. I. Twigg, Stefani N. Thomas

**Affiliations:** 1 Microbiology, Immunology, and Cancer Biology Graduate Program, University of Minnesota, Minneapolis, MN 55455, USA; perez888@umn.edu; 2 College of Biological Sciences, University of Minnesota, Minneapolis, MN 55455, USA; barre911@umn.edu; 3 Department of Laboratory Medicine and Pathology, University of Minnesota School of Medicine, Minneapolis, MN 55455, USA; twigg021@umn.edu

**Keywords:** high-grade serous ovarian cancer (HGSOC), mass spectrometry, proteomics, PARP inhibitors (PARPis)

## Abstract

High-grade serous ovarian cancer (HGSOC) is the most commonly diagnosed ovarian cancer subtype. Approximately half of all patients diagnosed with HGSOC are deficient in homologous recombination (HR), harbor *BRCA1/2* mutations, and are treated with poly (ADP-ribose) polymerase (PARP) inhibitors (PARPis). FDA-approved PARPis Olaparib, Niraparib, and Rucaparib all contribute to adverse effects in patients due to their poly-pharmacological properties. This feature necessitates investigation of global protein responses to PARPi treatment beyond DNA repair in the context of BRCA mutational status and HR deficiency. We sought to determine the landscape of differential PARPi-induced proteomes in HGSOC cells exhibiting different *BRCA1/2* mutational statuses. Here, we applied immunofluorescence microscopy to detect γH2AX, Rad51, and geminin foci as markers of DNA damage and repair upon treatment of HGSOC cells with IC_50_ doses of PARPis. Global proteome perturbations upon PARPi treatment were measured using quantitative mass spectrometry-based proteomics. The proteomic data highlighted cell line effects, masking high-dose PARPi treatment response. Interrogation of PARPi response within biological pathways identified through gene set enrichment analysis (GSEA) revealed significant changes to proteins involved in Epithelial–Mesenchymal Transition (EMT), E2F targets, and cholesterol homeostasis. Our study establishes proteomic evidence supporting the poly-pharmacological characteristics of Niraparib, Olaparib, and Rucaparib in HGSOC cells.

## 1. Introduction

Globally, ovarian cancer is the most lethal gynecologic malignancy. The four distinct histological subtypes of ovarian cancer are serous carcinoma (including high-grade and low-grade serous carcinoma), mucinous carcinoma, endometrioid carcinoma, and clear cell carcinoma [1,2,3]. High-grade serous ovarian cancer (HGSOC) is the most lethal and commonly diagnosed ovarian cancer subtype worldwide, with 42.97% of all ovarian cancer patients diagnosed with HGSOC in 2020 [4]. HGSOC is typically diagnosed at advanced stages with poor prognosis [5]. The standard of care for HGSOC treatment is an initial debulking surgery followed by first-line platinum-based chemotherapy with carboplatin and paclitaxel. Bevacizumab, a vascular endothelial growth factor (VEGF) inhibitor, is also typically given as first-line maintenance therapy [6]. Depending on primary chemotherapy response and *BRCA1/2* mutational status, targeted therapies such as poly (ADP-ribose) polymerase (PARP) inhibitors (PARPis) are commonly utilized for first-line maintenance therapy [7].

The molecular features of HGSOC are frequently characterized by mutations in *TP53*, leading to high genomic instability. Additionally, genetic alterations in *BRCA1* and *BRCA2* contribute to 30–40% of all HGSOC cases and lead to dysfunction in homologous recombination (HR) during DNA damage repair [8,9]. Importantly, HR deficiency, which is found in half of all HGSOC cases, encompasses mutations in other genes independent of aberrant *BRCA1/2* function including *RAD51C*, *RAD51D,* and *PALB2*, among others. The identification of these molecular features in HGSOC has influenced treatment strategies, specifically in therapeutically exploiting HR dysfunction within tumors using targeted therapies such as PARPis [8].

PARPis function by inhibiting PARP enzymatic activity during the repair of single-strand DNA breaks, and they are used to induce synthetic lethality in HGSOC with deficient HR or in tumors with *BRCA1/2* mutations. There are currently three FDA-approved PARPis utilized as maintenance therapy in HGSOC: Niraparib, Olaparib, and Rucaparib [10]. These PARPis share a benzamide core and differ in structure in their R-groups; this feature leads to differences in molecular size and flexibility. Although PARPi treatment has led to improvements in progression-free survival, little improvement is seen in overall survival due to the development of treatment resistance. Commonly observed mechanisms of PARPi resistance include, but are not limited to, restoration of HR through reversion mutations of key proteins involved in DNA damage repair, drug-efflux mechanisms, and replication fork stabilization [11].

Currently, Olaparib and Niraparib are the only PARPi treatments approved for frontline and recurrent maintenance therapy of HGSOC while Rucaparib is only approved for recurrent maintenance therapy [10]. Differences in PARPi treatments have been investigated in several studies, all highlighting distinct off-target mechanisms that are hypothesized to contribute to drug resistance [12,13,14]. IC_50_ doses are distinct across PARPis and PARP protein members. We have previously established distinct, off-target effects of low-dose Rucaparib and Niraparib treatment of HGSOC cells exhibiting *BRCA1* mutations [15]. Studies have highlighted differential PARPi effects on cancer cell proliferation and DNA damage repair, and selectivity to specific PARP enzymes [16,17,18]. Inhibition of kinases by differential PARPi treatment concentrations has been observed in cell lines [14].

Toward elucidating the poly-pharmacological effects of PARPis, here, we sought to determine the landscape of differential PARPi-induced proteomes in a panel of HGSOC cells exhibiting different *BRCA1/2* mutational statuses. Our global proteomic approach provides an unbiased analysis of PARPi-induced cellular pathway perturbations, beyond the well-documented DNA damage repair pathway.

## 2. Results

### 2.1. DNA Damage and Repair Are Distinctly Altered Following High-Dose PARPi Treatment in HGSOC Cells

The range of PARPi concentrations used to establish the IC_50_ dose was determined using the Genomics of Drug Sensitivity in Cancer (GDSC) database. GDSC determines IC_50_ doses of drugs using either a DNA dye (Syto60) or metabolic assays (Resazurin or CellTiter-Glo) to assess cell viability [19]. Here, we used Caov3, COV362, and PEO1 cells to represent HGSOC types with distinct *BRCA1/2* mutation statuses. Caov3 cells harbor functional *BRCA1/*2 genes, COV362 cells harbor a *BRCA1* missense mutation, and PEO1 cells contain a *BRCA2* missense mutation [20,21]. To assess the high-dose effects of individual PARPi treatments on Caov3, COV362, and PEO1 HGSOC cells, we first utilized the Sulforhodamine-B (SRB) assay to establish IC_50_ doses after 72 h (Supplementary Figure S1A). The SRB assay measures cell density following PARPi treatment, serving as a proxy in assessing cell viability. The IC_50_ dose of Olaparib was 3–10× higher across all cell lines compared to the Niraparib and Rucaparib IC_50_ doses: 10.68 µM in Caov3 cells, 80.68 µM in COV362 cells, and 109 µM in PEO1 cells. Conversely, the IC_50_ doses of Niraparib were consistently lower across the HGSOC cell panel: 1.55 µM, 8.53 µM, and 30.63 µM in Caov3, COV362, and PEO1 cells, respectively. Interestingly, *BRCA2*-mutated PEO1 cells exhibited the widest range in PARPi IC_50_ dose (78.37 µM) followed by *BRCA1*-mutated COV362 cells (72.15 µM). Caov3 cells, with functional *BRCA1/2*, had the narrowest range in PARPi IC_50_ dose (9.13 µM). Across our HGSOC cell line panel, cells required higher concentrations of Olaparib compared with Niraparib and Rucaparib to induce cell death (Supplementary Figure S1B).

**Figure 1:**
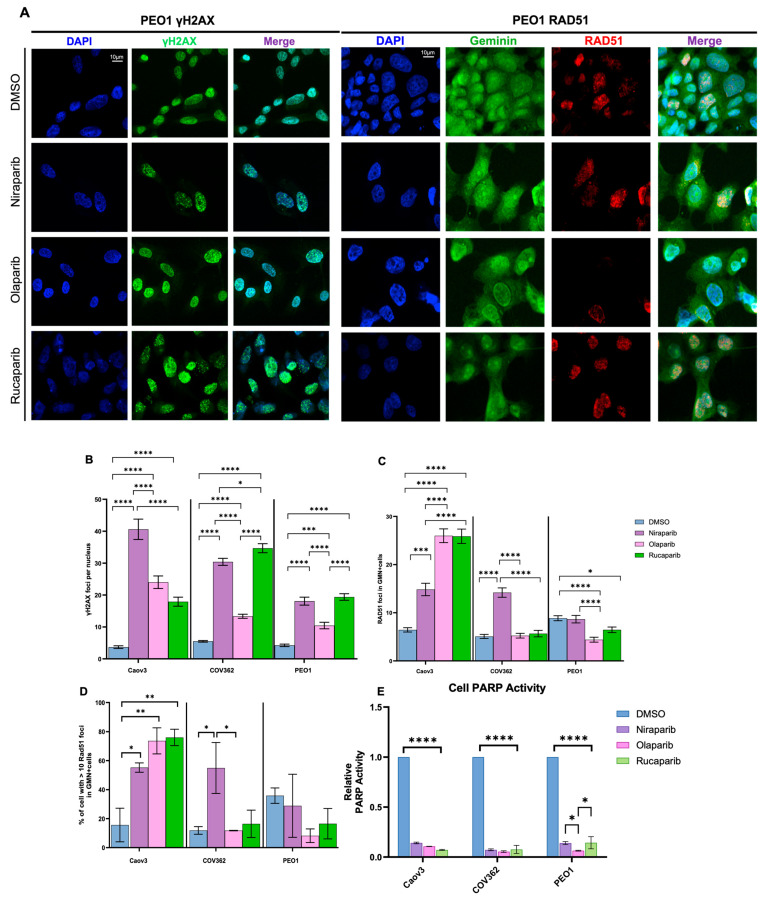

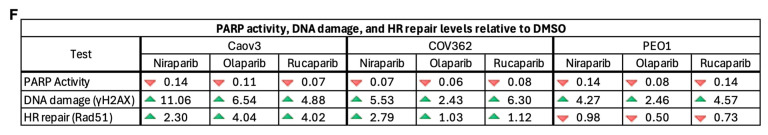
Response to PARPi treatment is drug- and cell line-dependent. Formation of γH2AX foci and RAD51 foci in geminin-positive cells was assessed using immunofluorescence microscopy following 72 h IC*50* treatment (**A**). γH2AX (**B**) and RAD51 (**C**) foci were quantified from *n* > 40 nuclei per experiment (*n* = 2 biological replicates). To determine HRD status in PEO1 cells, the total percent of cells with >10 RAD51 foci was quantified in *n* = 40 cells (**D**). PARP activity was assessed across HGSOC cell lines following treatment (**E**) (* *p* ≤ 0.05, ** *p* ≤ 0.01, *** *p* ≤ 0.001, **** *p* ≤ 0.0001). PARP activity, DNA damage, and HR levels relative to DMSO following PARPi treatment; arrows: red arrows indicate a decrease relative to DMSO and green arrows indicate an increase conpared to DMSO (**F**).

In addition to assessing changes to cell proliferation, we analyzed DNA damage and repair and PARP activity in PARPi-treated HGSOC cells. The formation of double-strand breaks (DSBs) in DNA was visualized by the presence of γH2AX foci upon PARPi treatment, while DNA damage repair was visualized by the formation of RAD51 foci in ≥40 geminin-positive cells (Figure 1A and Supplementary Figure S1C,D). Across all cell lines, PARPi treatment led to significant increases in γH2AX formation compared to DMSO (Figure 1B). Niraparib treatment was most effective in inducing DNA damage in Caov3 cells, followed by COV362 and PEO1 cells. High-dose Olaparib treatment resulted in a similar trend in γH2AX foci formation. Rucaparib treatment was most robust in inducing DSBs in COV362 cells compared to Caov3 and PEO1 cells. Among the three cell lines tested, PEO1 cells were the least responsive to PARPi treatment based on the induction of DSBs. RAD51 foci formation was highest in Caov3 cells and reduced in COV362 and PEO1 cells (Figure 1C). Olaparib and Rucaparib treatment resulted in similar levels of repair in both COV362 and PEO1 cells. Interestingly, high-dose Niraparib treatment led to greater RAD51 foci formation in cells with deficient *BRCA1* (COV362) and *BRCA2* (PEO1).

Next, HRD status for our HGSOC cell panel was determined by counting the percentage of cells containing >10 RAD51 foci in n = 40 geminin-positive cells (Figure 1D). Determination of HRD using immunofluorescence microscopy was adapted from the REcombination CAPacity (RECAP) test utilized by van Wijk et al. to determine HRD status on paraffin-embedded tissue [22]. Briefly, percentages of geminin-positive (GMN^+^) cells (n ≥ 40) with ≥5 RAD51 foci correspond to RECAP scores defined as follows: HRD ≤ 20%, HR-intermediate (HRI) 21–50%, and HR-proficient (HRP) > 50% [22]. Baseline levels of RAD51 foci in the DMSO-treated group indicate that PEO1 cells are HRI while Caov3 and COV362 cells are HRD (Figure 1D). Notably, treatment with Olaparib and Rucaparib in PEO1 cells decreased the overall percentage of cells with >10 RAD51 foci, characterizing these cells as HRD. Furthermore, PARPi treatment induced a significant increase in RAD51 foci in Caov3 cells, indicating HRP status following RECAP scoring guidelines. DNA damage response upon Olaparib and Rucaparib treatment in COV362 cells indicates HRD status; however, cell response to Niraparib treatment resulted in a RECAP score supporting HRI status. These results demonstrate that HR status, as determined by the RECAP test, is a dynamic characteristic upon PARPi treatment.

Following the assessment of DNA damage and repair, we next assessed enzymatic PARP activity across our HGSOC cell panel. All three PARPis significantly reduced PARP activity at their IC_50_ values compared with DMSO across all cells (Figure 1E). PARPi-specific differences were observed in PEO1 cells, where Olaparib treatment led to significantly reduced PARP activity compared to Niraparib and Rucaparib treatment. PARP activity, accumulation of DSBs, and HR repair are summarized in Figure 1F. Our results establish PARPi-specific differences contributing to distinct levels of DNA damage and repair in our HGSOC cell panel. Overall, we describe cell line- and PARPi-dependent differences through the assessment of cell viability, PARP activity, and the formation of damage and repair capacities upon IC_50_ treatment with PARPis.

### 2.2. High-Dose PARPi-Specific Effects Are Masked by HGSOC Cell Heterogeneity

To investigate the global proteomic response associated with high-dose PARPi treatment, HGSOC cells treated with PARPis were processed for quantitative mass spectrometry analysis. Pearson correlation analysis revealed that the proteomes of the biological replicates were highly correlated (range: 0.8–0.99) (Supplementary Figure S2A). Additionally, averaged protein abundances across cell lines and treatment conditions were approximately normally distributed prior to statistical analysis (Supplementary Figure S2B).

**Figure 2:**
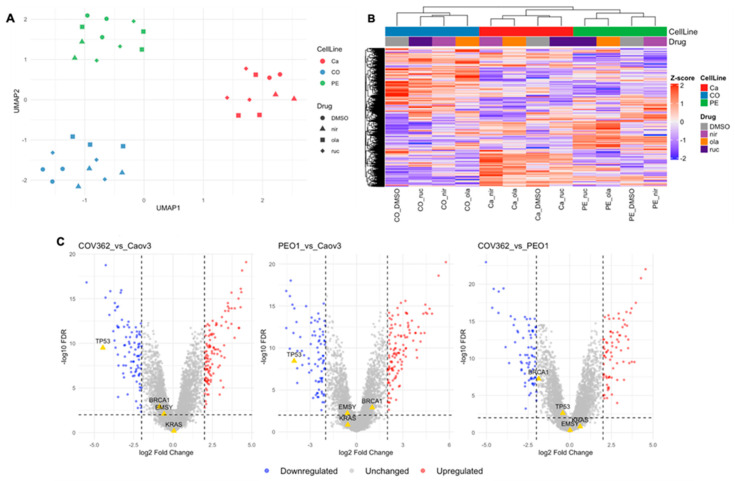
High-dose PARPi treatment induces significant HGSOC cell line-specific proteome perturbations. Caov3 (Ca), COV362 (CO), and PEO1 (PE) cells treated with PARPis or DMSO exhibit clustering based on cell type following UMAP analysis (**A**). Expression profiles illustrated by a heatmap reveal stronger cell line effects compared to high-dose PARPi-specific effects (**B**). Differential protein expression analysis conducted using PolySTest highlights significant proteome differences across cell lines; yellow triangles represent proteins that are characteristic of the HGSOC subtype (**C**).

Interestingly, our proteomic data exhibited strong cell line effects irrespective of PARPi treatment following UMAP analysis (Figure 2A). This finding was further validated upon analysis of expression profiles illustrated by heatmap analysis, where samples clustered primarily based on cell line (Figure 2B). Due to the strong presence of cell line effects dominating the clustering of our samples, we conducted differential protein expression analysis using PolySTest [23] against each cell line: COV362 vs. Caov3, PEO1 vs. Caov3, and COV362 vs. PEO1. Following differential protein analysis, we observed strong baseline proteomic differences across our HGSOC cell panel and highlighted prominent genomic markers that characterize HGSOC tumors (Figure 2C) [24,25]. Interestingly, TP53 protein abundances were significantly downregulated in COV362 and PEO1 cells compared to Caov3 cells while the protein abundances of HGSOC markers such as BRCA1, EMSY, and KRAS were largely the same across all three cell lines (Figure 2C).

### 2.3. Proteins Involved in Epithelial–Mesenchymal Transition and E2F Targets Are Significantly Perturbed in BRCA1-Mutated COV362 Cells upon PARPi Treatment

**Figure 3:**
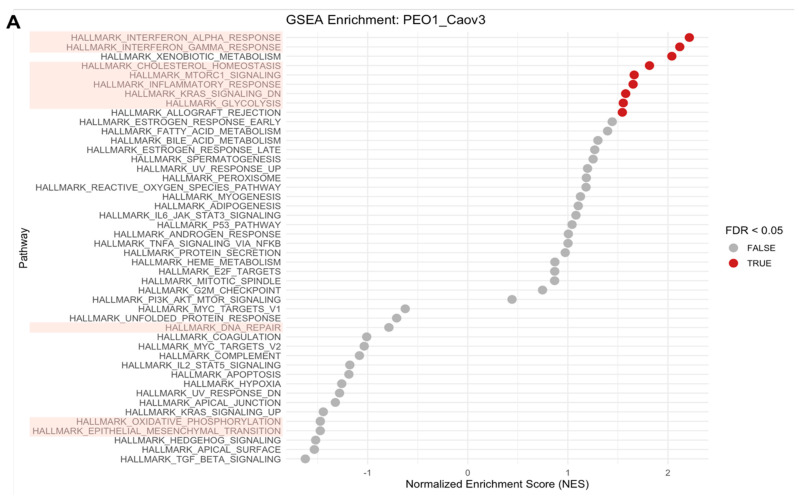

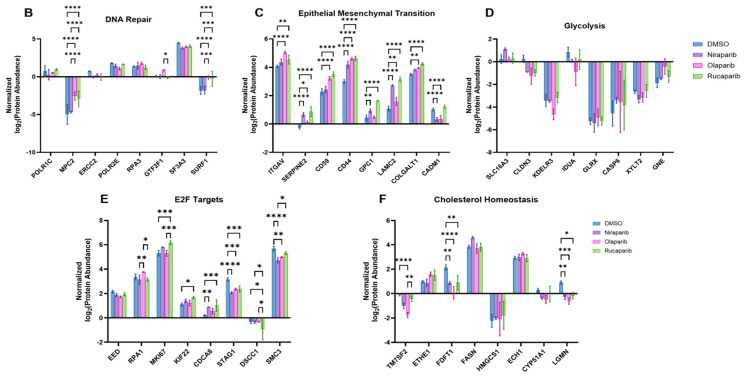
Gene set enrichment analysis of COV362 cells reveals enrichment of distinct biological pathways that are perturbed by high-dose PARPi treatment. Cell line effects were investigated between COV362 cells harboring a *BRCA1* mutation and Caov3 cells with functional *BRCA1/2* through gene set enrichment analysis (GSEA) (**A**). Biological pathways highlighted in pink are denoted as pathways of interest. Changes in protein abundances upon PARPi treatment were assessed in the top eight proteins driving pathway enrichment (**B**–**F**). Significant changes in protein abundances were assessed using two-way ANOVA followed by Tukey’s multiple comparison test (* *p* ≤ 0.05, ** *p* ≤ 0.01, *** *p* ≤ 0.001, **** *p* ≤ 0.0001).

Given the strong cell line effects observed within the proteomes of our HGSOC cell panel, we conducted gene set enrichment analysis (GSEA) of COV362 cells compared to Caov3 cells to identify the effects of PARPi treatments on biological pathways enriched in COV362 cells (Figure 3A and Supplementary Figure S3A,B). GSEA revealed a positive enrichment of glycolysis and cholesterol homeostasis, and a negative enrichment of pathways related to DNA repair, Epithelial–Mesenchymal Transition (EMT), and E2F targets. We next investigated the effects of high-dose PARPi treatments on the abundances of the top eight proteins driving enrichment of selected pathways. Surprisingly, high-dose PARPi treatment did not have a strong effect on protein abundances of the top eight proteins enriching for DNA repair (Figure 3B). The abundance of MPC2, a protein enriching for DNA repair upon PARPi treatment, was significantly increased following treatment with Olaparib and Rucaparib, but not Niraparib. This same trend was also observed for SURF1, which also plays a role in the functional enrichment of DNA repair, upon PARPi treatment. Interestingly, all top proteins enriching for EMT were altered following PARPi treatment in COV362 cells (Figure 3C). Although glycolysis was an enriched biological pathway in COV362 cells, PARPi treatments did not significantly perturb protein levels (Figure 3D). Top proteins enriching for E2F targets including MKI67, KIF22, CDCA8, STAG1, DSCC1, and SMC3 were significantly perturbed following PARPi treatment compared to the DMSO-treated control (Figure 3E). A significant decrease in protein abundances upon PARPi treatment was observed for proteins involved in cholesterol homeostasis including TM7SF2, FDFT1, and LGMN (Figure 3F). We also observed alterations in protein abundances for proteins involved in the G2M checkpoint (notably NOTCH2, YTHDC1, and RACGAP1) and MYC targets (specifically HK2 and SUPV3L1) (Supplementary Figure S3C,D).

### 2.4. The Proteome of BRCA2-Mutated PEO1 Cells Is Largely Unaffected by PARPi Treatment

**Figure 4:**
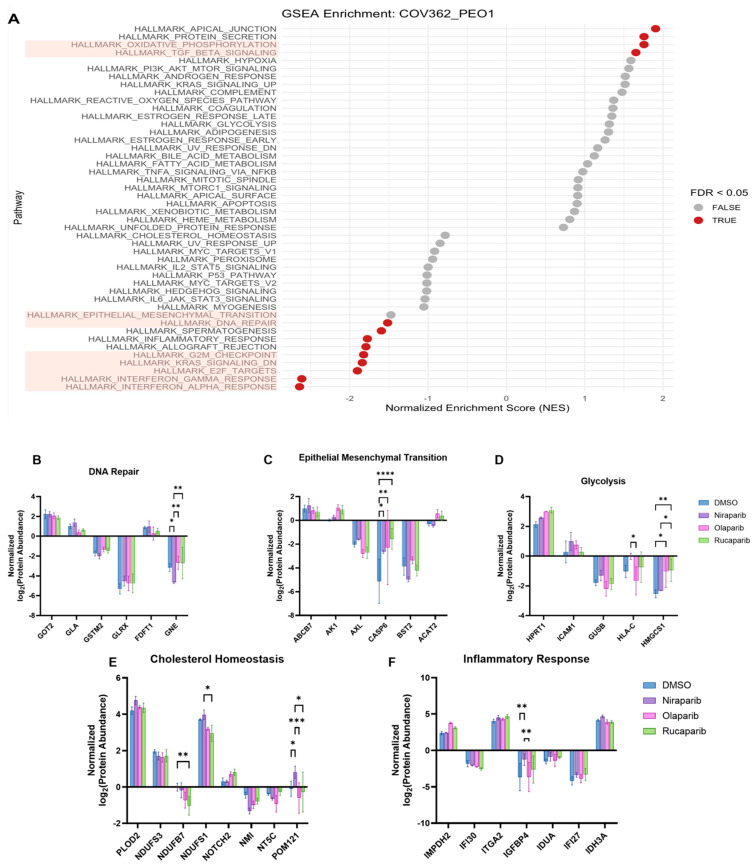
Proteome of BRCA2-mutated PEO1 cells exhibits minimal perturbations following high-dose PARPi treatment as evidenced by biological pathway enrichment. Cell line effects were investigated between PEO1 cells harboring a *BRCA2* mutation and Caov3 cells with functional BRCA1/2 through GSEA (**A**). Biological pathways highlighted in pink are denoted as pathways of interest. Changes in protein abundances upon PARPi treatment were assessed in the top proteins driving pathway enrichment (**B**–**F**). Significant changes in protein abundances were assessed using two-way ANOVA followed by Tukey’s multiple comparison test (* *p* ≤ 0.05, ** *p* ≤ 0.01, *** *p* ≤ 0.001, **** *p* ≤ 0.0001).

GSEA was conducted on PEO1 cells compared with Caov3 cells and revealed nine positively enriched pathways (Figure 4A and Supplementary Figure S4A,B). Given the prominent role of PARPis in DNA damage response, we interrogated PARPi effects in the top proteins driving pathway enrichment of DNA repair in PEO1 cells (Figure 4B). GNE was the only protein perturbed by high-dose Niraparib treatment. Next, proteins distinctly enriching for EMT, glycolysis, and cholesterol homeostasis were also assessed in PEO1 cells given their observed enrichment in COV362 cells. CASP6 abundance was significantly increased following treatment with Niraparib, Olaparib, and Rucaparib compared to DMSO (Figure 4C). For the top proteins enriching for glycolysis, only HMGCS1 abundance was elevated after Olaparib and Rucaparib treatment (Figure 4D). We observed a decreasing trend in NDUFS3 abundance following PARPi treatment; however, statistical significance was achieved only after Rucaparib treatment (Figure 4E). Additionally, within the inflammatory response pathway, IGFBP4 abundance was significantly elevated upon Niraparib treatment (Figure 4F). Among the top proteins identified in the positively enriched pathways identified through GSEA (Supplemental Figure S4C–G), LGMN abundance was significantly decreased following Olaparib and Rucaparib treatment (Supplemental Figure S4E).

### 2.5. GSEA Between BRCA1- and BRCA2-Mutated Cell Lines Identifies Enrichment of Biological Pathways That Are Strongly Perturbed by PARPi Treatment

**Figure 5:**
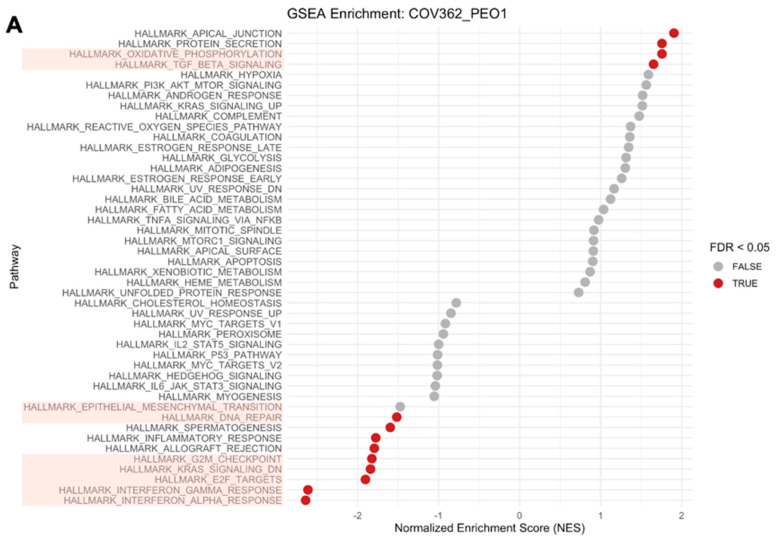

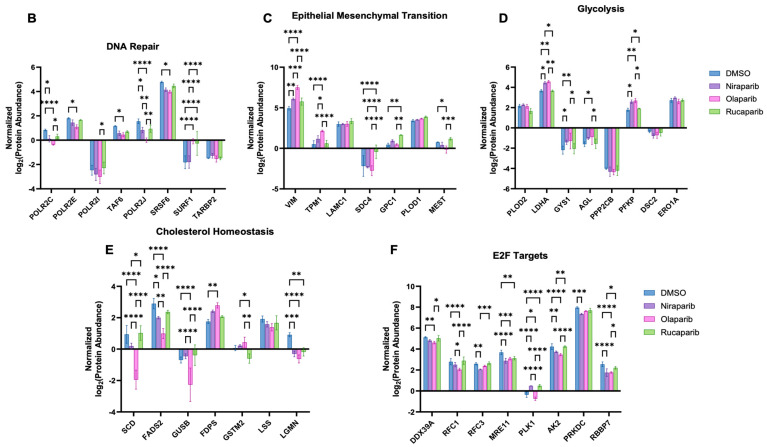
Interrogation of cell line effects between COV362 and PEO1 cells demonstrates robust PARPi response in specific biological pathways identified through GSEA. GSEA was performed between COV362 and PEO1 cells. Pathways highlighted in pink represent biological processes of interest (**A**). Perturbations to protein abundances upon PARPi treatments were assessed in COV362 cells for the top proteins driving pathway enrichment (**B**–**F**). Significant changes in protein abundances were assessed using two-way ANOVA followed by Tukey’s multiple comparison test (* *p* ≤ 0.05, ** *p* ≤ 0.01, *** *p* ≤ 0.001, **** *p* ≤ 0.0001).

GSEA was previously conducted on COV362 and PEO1 cells compared with Caov3 cells (Figure 3 and Figure 4) to investigate biological pathways driving sample clustering between cell lines with dysfunctional BRCA1/2 (COV362 and PEO1) compared with functional BRCA1/2 (Caov3). A similar GSEA approach was achieved to compare proteome perturbations following PARPi treatment between a *BRCA1*-mutated cell line (COV362) and a *BRCA2*-mutated cell line (PEO1) (Figure 5A). In this analysis, COV362 cells were positively enriched for pathways including oxidative phosphorylation and TGF-β signaling, and negatively enriched for DNA repair, the G2M checkpoint, KRAS signaling, E2F targets, interferon gamma response, and interferon alpha response. Among the top proteins enriching for DNA repair, we observed significant decreases in abundance for POLR2C, POLR2J, and SURF1 after treatment with PARPis (Figure 5B). Five of the seven top proteins enriching for EMT were significantly altered upon PARPi treatment; Niraparib and Olaparib induced significant increases in VIM, while protein levels of TPM1 were increased in the Rucaparib-treated sample (Figure 5C). In the top proteins enriching for glycolysis, LDHA and PFKP were increased after treatment with Niraparib and Olaparib (Figure 5D). The proteins involved in cholesterol homeostasis also demonstrated changes in abundance as a result of PARPi treatment including SCD, FADS2, and LGMN (Figure 5E). All top eight proteins enriching for E2F targets demonstrated some level of protein alteration upon drug treatment (Figure 5F). Notably, the abundances of proteins such as ITGB3, CD55, and PARP14 were also perturbed in PARPi-treated samples (Supplementary Figure S5C,D). Despite KRAS signaling and TGF-β signaling being enriched biological pathways following GSEA, few proteins were significantly affected by treatment (Supplementary Figure S5E,F).

### 2.6. Proteomic Analysis of PARPi-Treated HGSOC Cells Reveals Poly-Pharmacological Properties of Drug Treatment Beyond DNA Repair

We next assessed protein alterations in Caov3 cells upon PARPi treatment in biological pathways identified through the GSEA generated from COV362 and PEO1 cells (Supplementary Figure S6). Protein abundances were largely unaffected by PARPi treatment in Caov3 cells.

For each pathway identified through GSEA across all cell lines, proteins that enriched for distinct biological pathways were isolated in a separate analysis that interrogated protein–protein interaction (PPI) using STRING-db [26].

**Figure 6:**
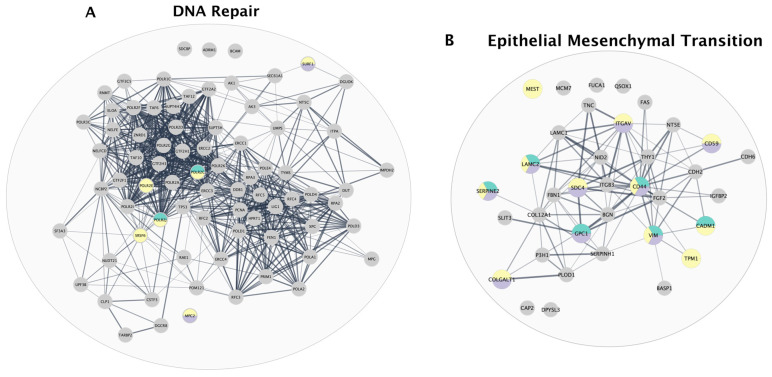

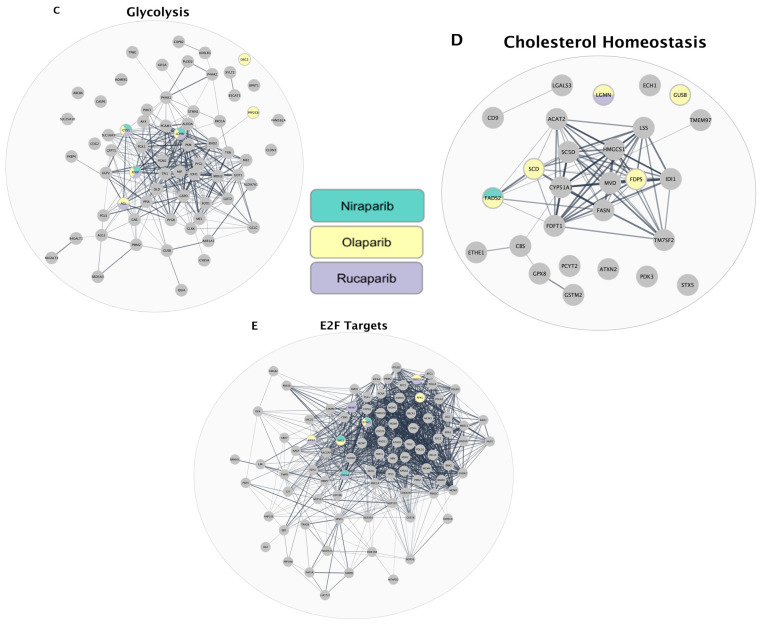
PARPi treatment perturbs protein abundances across distinct biological pathways. Pathway enrichment analysis identified biological processes that distinctly characterize the proteomes of HGSOC cell lines. Protein–protein interaction networks were developed from core enriched genes identified through GSEA including (**A**) DNA repair, (**B**) Epithelial Mesenchymal Transition, (**C**) Glycolysis, (**D**) Cholesterol Homeostasis, and (**E**) E2F Targets using STRING-db. Proteins significantly perturbed by PARPi treatment are highlighted.

Next, proteins that demonstrated a significant change in abundance compared with DMSO upon PARPi treatment were highlighted in each PPI network, resulting in annotated PPI networks showing proteins that are commonly perturbed by at least one PARPi per pathway (Figure 6 and Supplementary Figure S7). Surprisingly, the DNA repair protein network highlighted six proteins perturbed by PARPi treatment: POLR2C and POLR2J are perturbed by Niraparib and Olaparib, SURF1 and MPC2 by Olaparib and Rucaparib, and POLR2E and SRSF6 by Olaparib (Figure 6A). Within the EMT protein network, SERPINE2, LAMC2, CD44, and VIM protein abundances are all affected by Niraparib, Olaparib, and Rucaparib treatment (Figure 6B). All three PARPi treatments consistently perturbed GYS1, PFKP, and LDHA levels, highlighted in the glycolysis PPI network (Figure 6C). The PPI network for cholesterol homeostasis illustrates distinct PARPi-specific responses, with three proteins altered by Olaparib, one protein altered by Niraparib and Olaparib, and another protein altered by Olaparib and Rucaparib (Figure 6D). Within the PPI network enriching for E2F targets, STAG1 levels were perturbed by all three PARPis (Figure 6E). Our results demonstrate poly-pharmacological effects of Niraparib, Olaparib, and Rucaparib beyond DNA repair.

## 3. Discussion

Here, we sought to investigate cell-wide proteomic perturbations upon Niraparib, Olaparib, or Rucaparib treatment in HGSOC cells. The cell panel used in this study harbors *TP53* mutations, which is characteristic of HGSOC; importantly, the cell lines harbor distinct *BRCA1/2* mutation statuses [21]. Given the poly-pharmacological properties reported for PARPis, highlighting distinct, off-target binding to kinases and other molecules, there is an unmet need to characterize the global effects of PARPis at the level of the proteome [14,18,27]. Our results highlight changes in protein abundances in biological pathways pertaining to EMT, cholesterol homeostasis, and E2F targets.

Due to variations in IC_50_ concentrations across cell viability assays in published studies, we empirically determined the IC_50_ dose of Niraparib, Olaparib, and Rucaparib in an HGSOC cell line panel representing distinct *BRCA1/2* mutational statuses [28,29,30]. Our results demonstrate that, across our cell panel, HGSOC cells required a higher dose of Olaparib to induce cell death. Additionally, the IC_50_ dose of Olaparib in PEO1 cells significantly reduced PARP activity compared with Niraparib and Rucaparib. Although differences in response to Olaparib are cell line-dependent, PEO1 cells harbor a nonsense mutation in exon 11 of *BRCA2* [31]; this genomic feature has been shown to render ovarian cancer patients more sensitive to Olaparib treatment [32].

The poly-pharmacology of PARPis is proposed to occur at three levels: (1) inter-family protein binding, (2) intra-family protein binding, and (3) multi-signaling activities mediated by the same protein target [33]. At the level of inter-family protein binding, Olaparib was found to not influence kinase activity; however, Rucaparib presented multitarget kinase inhibitor activity at low micromolar ranges [34]. Furthermore, Rucaparib and Niraparib have been previously shown to have large intra-family protein binding profiles affecting key enzymes: Rucaparib inhibits Hexose-6-phosphate dehydrogenase (H6PD) activity while Niraparib inhibits Deoxycytidine kinase (DCK) [35]. Differing PARP trapping efficiencies across PARPi treatments are thought to contribute to differences in PARP-1 auto-PARylation, further highlighting another facet of the poly-pharmacology of these drugs [36].

PARPi potency has been previously investigated in triple-negative breast cancer cells, where higher potencies of Rucaparib and Olaparib treatment were associated with G(2)/M arrest [16]. Our data demonstrates a positive enrichment of the E2F pathway, which regulates G2 arrest, in PEO1 cells. Conversely, COV362 and Caov3 cells exhibited a negative enrichment of the E2F pathway, indicating a downregulation of this biological process [37]. Within the proteins enriching for the E2F pathway, we observed significant changes to KIF22 abundance upon treatment with Rucaparib and Olaparib, but not Niraparib, across all cell lines (Figure 6E). KIF22 has previously been shown to associate with other mitotic proteins such as TPX2 at stalled replication forks with PARP1, facilitating DNA repair [38]. Our findings indicate that PARPi potency of Niraparib, Olaparib, or Rucaparib distinctly alters the abundance of proteins that interact with PARP1. Additionally, the inhibition of STAG1, a protein involved in the E2F pathway, has been shown to sensitize HeLa and HepG2 cells to PARPis [39]. Interestingly, we observed the EMT pathway to be negatively enriched in COV362 cells; however, upon treatment with PARPis, proteins involved in EMT trended toward an increase in abundance compared to the control in COV362 cells (Figure 3). In support of this finding, DNA damage was previously shown to induce EMT in cancer cells via interactions between PARP1 and ALC1; induction of EMT led to increased DNA damage repair through HR [40,41]. Our global proteome analysis potentially highlights proteins that not only are off-targets of PARPi treatment but may also contribute to PARPi resistance. These findings can facilitate the identification of novel protein targets to sensitize tumor cells to PARPi treatment.

Importantly, the poly-pharmacology of PARPis has profound effects in the clinic by contributing to suspected adverse drug reactions (ADRs). Rucaparib treatment has been found to have the highest number of ADRs, followed by Niraparib [13]. Overall, our results emphasize the poly-pharmacological characteristics of Niraparib, Olaparib, and Rucaparib in HGSOC.

Mass spectrometry-based proteomic analysis of HGSOC cells treated with high-dose PARPis induced cell line-specific proteomic perturbations (Figure 2). Our results, utilizing an unbiased characterization of the proteomic landscape of ovarian cancer cells, illustrate HGSOC heterogeneity in both baseline cell characteristics and treatment response to Niraparib, Olaparib and Rucaparib. Importantly, we observed strong proteomic differences between Caov3, COV362, and PEO1 cells despite minor changes in protein abundances of HGSOC markers including EMSY, KRAS, and BRCA1 [24,25]. Notably, the effects of high-dose treatment of PARPis were masked by prominent cell line effects. This high level of heterogeneity is a prominent feature in HGSOC, contributing to differences in patient treatment response and resistance to therapy [42,43]. Given that cell line effects dominated the proteomes of our HGSOC cell panel, we conducted GSEA to identify biological pathways enriched at baseline and assessed the effects of high-dose PARPi treatment on the abundances of proteins enriching for a specific biological pathway. Our findings establish COV362 cells to be most sensitive to drug treatment, while PEO1 and Caov3 cells were least responsive to protein perturbations associated with high-dose PARPi treatment. Moreover, our study demonstrates that the effects of PARPi treatment on the proteome are influenced by cellular heterogeneity. Broadly, patient stratification for PARPi treatment would benefit from taking into account factors beyond *BRCA1/2* mutation status to improve treatment response.

One limitation of this study is that differences in proteomes across distinct cell lines cannot directly be attributed to HR or *BRCA1/2* mutational status. Moreover, IC_50_ doses of PARPi treatments vary depending on cell line type, duration of drug treatment, and methodology [44,45,46]. However, our data demonstrates that high-dose PARPi treatment results in distinct global changes to protein abundances beyond its primary role in inducing DNA damage. Interestingly, many proteins associated with DNA repair were not changed upon PARPi treatment; nonetheless, protein activity could be altered by PARPi treatment.

## 4. Materials and Methods

### 4.1. Cell Culture Conditions and Lysate Preparation

COV362 (European Collection of Authenticated Cell Cultures, ECACC, Porton Down, Salisbury, UK) and Caov3 cells (American Type Culture Collection, ATCC, Manassas, VA, USA) were incubated at 37 °C in 5% CO_2_ in Dulbecco’s Modified Eagle Medium (DMEM) supplemented with 10% fetal bovine serum (FBS), 1% penicillin–streptomycin, and 100 µg/mL Normocin. PEO1 cells (ECACC) were cultured under the same conditions in RPMI-1640 medium supplemented with 10% FBS, 2 mM sodium pyruvate, 1% penicillin–streptomycin, and 100 µg/mL Normocin. Caov3, COV362, and PEO1 cells were seeded at a density of 3 × 10^6^ cells in 10 cm dishes prior to treatment for 72 h with IC_50_ dose of a PARP inhibitor (Niraparib, Olaparib, or Rucaparib purchased from Selleck Chemical, Houston, TX, USA) dissolved in dimethyl sulfoxide (DMSO). All conditions were prepared with n = 3 biological replicates, except for COV362 cells treated with Olaparib, where n = 2 replicates were obtained. After treatment, cells were washed with ice-cold phosphate-buffered saline (PBS) and collected from each dish using a cell scraper, then centrifuged at 2500× *g* at 4 °C for 5 min, washed with PBS, and centrifuged once more. The cell pellets were stored at −80 °C.

### 4.2. Determination of IC_50_ PARPi Dose Using Sulforhodamine-B (SRB) Assay

The Sulforhodamine-B (SRB) assay was used to establish the half-maximal inhibitory concentration (IC_50_) dose following protocols adapted from Voigt et al. and Vichai et al. [47,48] To determine the IC_50_ dose for Niraparib, Olaparib, and Rucaparib, cells were seeded into 96-well plates in the following densities: Caov3 cells, 12,800 cells/well; COV362 cells, 6400 cells/well; and PEO1 cells, 12,800 cells/well. All cell lines were seeded in n ≥ 3 wells per condition for 24 h prior to PARPi treatment for 72 h. COV362 and Caov3 cells were treated with 0–320 µM of Niraparib, Olaparib, or Rucaparib in DMSO. PEO1 cells were treated with 0–460 µM of PARPi. Ranges of treatment doses for all cell lines were determined using the Genomics of Drug Sensitivity in Cancer database [19]. After treatment, the cell medium was discarded, and cells were washed with 200 µL of PBS and fixed in 100 µL of cold 10% trichloroacetic acid (TCA) solution (Sigma Aldrich, St. Louis, MO, USA) at 4 °C for 1 h. Following fixation, cells were rinsed with deionized water (200 µL/well, five washes) and stained with 100 µL of 0.4% SRB solution (200 mg SRB dissolved in 1% acetic acid) for 30 min at room temperature. After staining, cells were washed with 1% acetic acid (200 µL/well, five washes) and left to air-dry for at least 24 h prior to dissolving bound SRB in 100 µL/well of 10 mM Tris Base for 10 min with gentle shaking. The optical density (OD) was then measured using an automated 96-well plate reader (Synergy HTX multi-mode reader, BioTek, Winooski, VT, USA) at a wavelength of 564 nm. To determine % cytotoxicity, the OD of the DMSO control was subtracted from the OD of the treatment and divided by the OD of the DMSO control.

### 4.3. PARP Activity Assay

PARP enzymatic activity was assessed in Caov3, COV362, and PEO1 cells after IC_50_ PARPi treatment according to the manufacturer’s instructions for adherent cells (4672-096-K R&D Systems, Minneapolis, MN, USA ). Briefly, cells treated with IC_50_ doses of PARPi were washed with ice-cold 1X PBS and harvested via cell scraping from 15 cm plates prior to centrifugation at 200× *g* for 10 min at 4 °C. Cell pellets were resuspended in 10× pellet volume of 1X PARP buffer containing 1% Triton X-100, 1:100 protease inhibitor, 0.4 mM phenylmethylsulfonyl fluoride (PMSF), and 0.4 M NaCl. Cell suspensions were incubated on ice for 30 min with occasional vortexing followed by centrifugation at 10,000× *g* for 20 min at 4 °C to pellet insoluble material. Cell supernatants were collected, and protein concentrations were determined using the Bradford assay. Then, 1X PARP cocktail, 1X PARP buffer, and 20 µg of protein from each cell line and PARPi treatment were added to each well (n = 3 wells per cell line, per treatment condition) in a 96-well plate and incubated for 60 min at room temperature. After incubation, the plate was washed with 1X PBS (200 µL/well, 4 times), and Strep-HRP was added to each well followed by incubation for 20 min at room temperature and washing with 1X PBS. TAC-Sapphire was added to each well, incubated for 30 min in the dark prior to the addition of 0.2 M HCl, and read in a plate reader at 450 nm.

### 4.4. γH2AX, Rad51, and Geminin Foci Detection

Caov3, COV362, and PEO1 cells were treated with IC_50_ dose of a PARPi (Niraparib, Olaparib, or Rucaparib) for 72 h. Cells were fixed on Poly-D-lysine-coated (A3890401 Gibco, Waltham, MA, USA) coverslips in 4% formaldehyde in DPBS (14-190-250 Thermo Scientific, Waltham, MA, USA) and incubated at room temperature for 5 min. Following fixation, cells were washed twice with PBS and permeabilized with 0.1% Triton X-100 for 15 min. After permeabilization, cells were washed three times for 5 min each with PBS. Next, non-specific binding sites were blocked with blocking buffer (3% BSA, 0.5% fish gelatin, 0.01% Tween20 in PBS) for 30 min and incubated with primary mouse monoclonal antibody against γH2AX (05-636, Millipore Sigma, Burlington, MA, USA), geminin (sc-74456, Santa Cruz Biotechnology, Santa Cruz, CA, USA), or primary rabbit monoclonal antibody against Rad51 (88755, Cell Signaling Technologies, Danvers, MA, USA) diluted in blocking buffer (1:500) for 1 h at room temperature. After immunostaining with primary antibodies, cells were washed three times for 5 min with PBS prior to incubation in goat anti-mouse-488 secondary antibody (A32723TR, Invitrogen, Carlsbad, CA, USA) or goat anti-rabbit-647 secondary antibody (A32733TR, Invitrogen, Carlsbad, CA, USA) at a dilution of 1:1000 in blocking buffer for 1 h at room temperature. Nuclei were counterstained with DAPI (D9542, Sigma-Aldrich). The cells were imaged at 40× objective using a Nikon AX R confocal microscope (Nikon Instruments, Melville, NY, USA) equipped with 405, 488, and 640 nm lasers. For the detection of γH2AX foci, the following filters were used: DAPI at 420–476 nm and AF488 at 503–541 nm. For the detection of Rad51 foci and geminin, the following filters were used: DAPI at 429–474 nm, AF488 at 503–541 nm, and AF647 at 653–726 nm. Balanced acquisition was utilized for detection. At least 40 cells per cell line, per PARPi treatment, were preprocessed in Fiji using mean filter (radius = 2 pixels), and foci were detected using the Find Maxima function with prominence parameter adjustment to minimize background signal.

### 4.5. Protein Digestion and TMT Labeling

Cell pellets were resuspended in urea lysis buffer (8 M urea, 75 mM NaCl, 50 mM Tris pH 8.0, 1 mM EDTA, 2 µg/mL aprotinin, 10 µg/mL leupeptin, 1 mM PMSF), vortexed for 10 s, and incubated on ice for 15 min, followed by another 10 s vortex and another 15 min incubation on ice. After 20,000× *g* centrifugation at 4 °C for 10 min, the supernatant was transferred to a new tube and the protein concentration was determined with a BCA protein assay.

For digestion, 110 µg of protein per sample was used for disulfide bond reduction with 50 mM dithiothreitol (DTT) for 1 h at 37 °C and then alkylated with 100 mM iodoacetamide (IAA) for 45 min at room temperature in the dark. The samples were diluted 1:4 with 50 mM Tris (pH 8.0) and subsequently digested with Lys-C at an enzyme/substrate ratio of 1:50 for 2 h with shaking at room temperature, followed by trypsin digestion at an enzyme/substrate ratio of 1:50 overnight with shaking at room temperature. The digestion reactions were acidified and quenched with 10% formic acid to a final concentration of 0.1% followed by centrifugation at 1500× *g* for 15 min at room temperature.

The peptide samples were then desalted using 1cc C18 SepPak cartridges with a vacuum manifold (Waters, Milford, MA, USA). The eluates were dried using vacuum centrifugation and reconstituted in 50 mM HEPES pH 8.5 prior to the measurement of peptide concentration with a BCA protein assay. The peptide samples were labeled with the TMT-10plex^TM^ Label Reagent set according to the manufacturer’s protocol (90110, Thermo Fisher Scientific, Waltham, MA, USA). Samples were multiplexed into four sets of TMT-10plex as highlighted in Supplementary Table S1. Each TMT label was reconstituted in 20 µL of anhydrous acetonitrile and added to 25 µg of peptide per sample for 1 h at room temperature and then pooled, desalted, and dried using vacuum centrifugation.

### 4.6. High-pH RPLC Offline Fractionation

The TMT-labeled samples were resuspended in Buffer A (20 mM ammonium formate pH 10 in 98:2 water/acetonitrile) prior to high-pH reversed-phase high-performance liquid chromatography (HPLC) fractionation. Samples were fractionated using offline high-pH reversed-phase liquid chromatography (bRP-HPLC) where 96 fractions were prepared at a flow rate of 0.2 mL/min with the following gradient: 0% Solvent B for 7 min, 0–16% Solvent B at 7–13 min, 16–40% Solvent B at 13–73 min, 40–44% Solvent B at 73–77 min, and 44–60% B at 77–96 min. Fractions were then pooled into 24 fractions using the pooling fraction scheme outlined in Mertins et al. [49]. Twenty-four bRP-HPLC fractions from each sample set were subsequently dried and reconstituted in 5% ACN/0.1% FA.

### 4.7. MS Analysis

Liquid chromatography–tandem mass spectrometry (LC-MS/MS) analysis was performed using a Vanquish Horizon liquid chromatography system coupled with an Orbitrap Q Exactive Plus mass spectrometer (Thermo Fisher, Waltham, MA, USA). The samples were reconstituted in 2% acetonitrile and 0.1% formic acid and injected into an Accucore RP-MS HPLC Column (Thermo Scientific, Waltham, MA, USA, Column Format = Analytical, I.D. × L = 2.1 × 100 mm, Particle Size = 2.6 µm, Pore Size = 80 Å) with the spray voltage set to 1.6 kV. Peptides were separated using a gradient of Buffer A (water and 0.1% formic acid) and Buffer B (acetonitrile and 0.1% formic acid) at a flow rate of 0.5 mL/min. The gradient was as follows: 0–0.5 min 5% B, 0.5–60.5 min 5-45% B, 60.5–61.5 min 45–95% B, 61.5–61.6 min 95-5% B, and 61.6–63 min 5% B. Data were acquired using a top 10 DD-MS^2^ method. MS1 scans were acquired in the orbitrap with a scan range of 350–1800 *m*/*z* at a resolution of 35,000, and ions with charges 2+ to 7+ were selected for Collision-Induced Dissociation (CID)-based MS/MS fragmentation. Dynamic exclusion duration was set to 4 s. MS2 fragmentation was conducted using Higher-energy Collision Dissociation (HCD) with a scan range of 200–2000 *m*/*z* and a resolution of 35,000.

### 4.8. Proteomic Data Analysis

Raw mass spectrometry data were processed using MaxQuant version 2.1.4 on the Galaxy-P platform. Raw files were processed by setting group-specific parameters for enzymatic digestion, variable modifications, fixed modifications, and reporter ion MS2. Enzymatic digestion was set to specific with Trypsin/P and LysC with an allowable max number of missed cleavages set at 2. Oxidation of methionine and acetylation of protein N-terminus were set as variable modifications, and cysteine carbamidomethylation was set as a fixed modification. For TMT-labeled protein quantification, reporter ion MS2 was selected and isobaric labels with their correction factors were applied from a TMT 10-plex and multiplexed into four sets: 126, 127N, 127C, 128N, 128C, 129N, 129C, 130N, 130C, and 131. All other parameters within MaxQuant remained under default settings. A *Homo sapiens* Swiss-Prot database (downloaded on 13 November 2023) was used for protein identification. All subsequent data analyses were performed using Perseus version 2.0, RStudio (ver. 2025.09.0+387), gene set enrichment analysis (GSEA), and STRING-db. Data from the ProteinGroups.txt file output from the database search conducted on MaxQuant was subsequently filtered and prepared using Perseus. Proteins only identified by a modification site, proteins identified as a potential contaminant from the CRAPome database [50], and proteins identified by their reverse sequence (decoys) were removed from the dataset. Data were log_2_-transformed followed by median normalization, and protein hits containing 2/3 valid values per sample group (biological replicates) were retained in the dataset with the remaining nonvalid values imputed by QRILC. ComBat was used to adjust for batch effect in the dataset. Outliers were identified and removed (PE_nir_1). A total of 5228 proteins were quantified across all samples.

Given the strong cell line effects driving sample clustering, PolySTest was used to identify significantly dysregulated proteins between cell types irrespective of PARPi treatment based on FDR < 0.05 and a log_2_(fold-change) cutoff of ≤−2 and ≥2. Next, to identify biological pathways driving cell line effects, gene set enrichment analysis (GSEA) was conducted using a pre-ranked gene list generated from the protein abundance data for the following comparisons: COV362 vs. Caov3, PEO1 vs. Caov3, and COV362 vs. PEO1. Protein abundances for the top N proteins driving pathway enrichment were then assessed for perturbations upon PARPi treatment using two-way ANOVA followed by Tukey’s multiple comparison test. Protein–protein interaction (PPI) networks were generated for the top N proteins driving pathway enrichment using STRING-db ver 12.0 (string-db.org) with the following parameters: protein–protein interaction networks were filtered for interactions with medium confidence (0.400). PPI networks were exported on Cytoscape ver. 3.10.3, where protein levels significantly changed by PARPi treatment were highlighted.

## 5. Conclusions

Using an unbiased, mass spectrometry-based proteomic approach, we established global perturbations to the proteomes of HGSOC cell lines treated with high-dose PARPis (Niraparib, Olaparib, and Rucaparib). Our results highlight poly-pharmacological characteristics of PARPis affecting protein abundances across biological pathways beyond DNA repair including EMT, E2F targets, cholesterol homeostasis, and glycolysis. Additionally, our results demonstrate the effects of cell line heterogeneity on PARPi response, emphasizing that off-target effects of PARPis occur distinctly across HGSOC cells.

## Data Availability

The mass spectrometry proteomic data have been deposited in the ProteomeXchange Consortium via the PRIDE [51] partner repository (https://www.ebi.ac.uk/pride/ (accessed on 1 November 2025)) with the dataset identifier PXD067886.

## Supplementary Materials

The following supporting information can be downloaded at https://www.mdpi.com/article/10.3390/ijms262411820/s1.

## Abbreviations

The following abbreviations are used in this manuscript:

ADRs	Adverse drug reactions
EMT	Epithelial–mesenchymal transition
GDSC	Genomics of Drug Sensitivity in Cancer
GSEA	Gene set enrichment analysis
HGSOC	High-grade serous ovarian cancer
HR	Homologous recombination
HRD	Homologous recombination deficiency
HRI	Homologous recombination-intermediate
HRP	Homologous recombination-proficient
PARP	Poly (ADP-ribose) polymerase
PPI	Protein–protein interaction
RECAP	Recombination CAPacity
SRB	Sulforhodamine-B
