# Supplementary material for: Global Proteomic Determination of the Poly-Pharmacological Effects of PARP Inhibitors Following Treatment of High-Grade Serous Ovarian Cancer Cells
Source: Int J Mol Sci. 2025 Dec 7;26(24):11820. doi: 10.3390/ijms262411820 (PMC12733200; doi:10.3390/ijms262411820)
Supplement: Supplementary file 1 [file ijms-26-11820-s001.zip › ijms-3995780-supplementary.pdf]

A

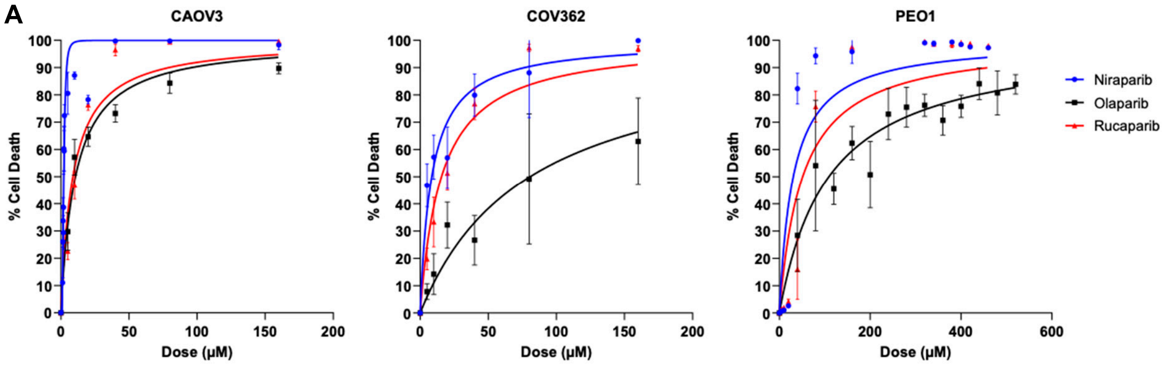

B

Cell line	PARPi IC_{50} (μM)		
	Olaparib	Niraparib	Rucaparib
Caov3 (*BRCA* WT)	10.68	1.55	8.82
COV362 (*BRCA1* Mut)	80.68	8.53	15.66
PEO1 (*BRCA2* Mut)	109	30.63	51.24

C

Caov3 γH2AX

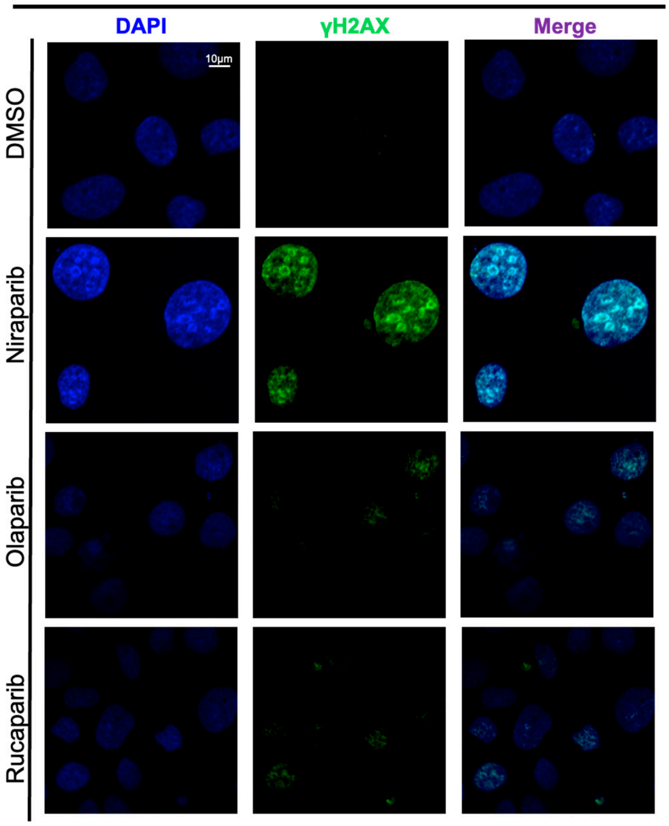

D

COV362 γH2AX

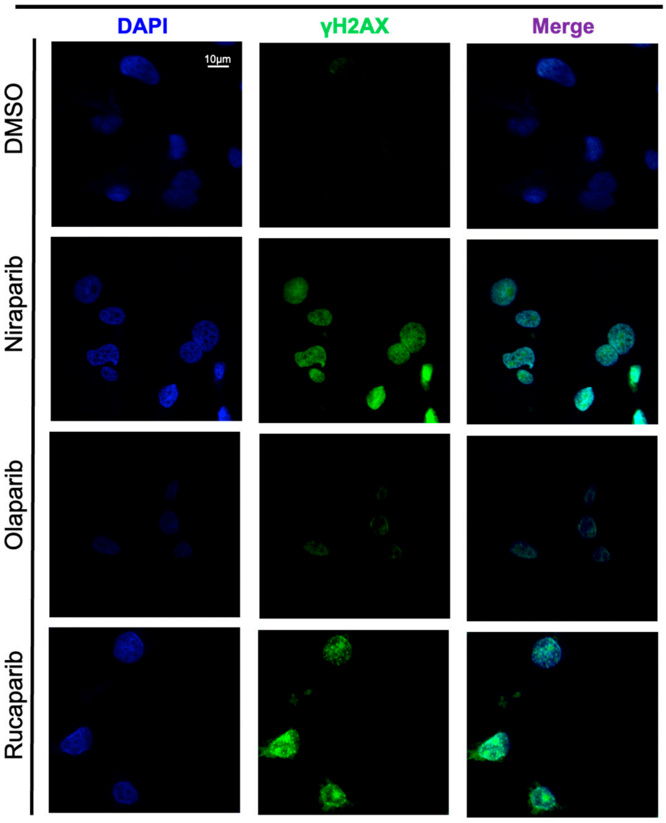

E

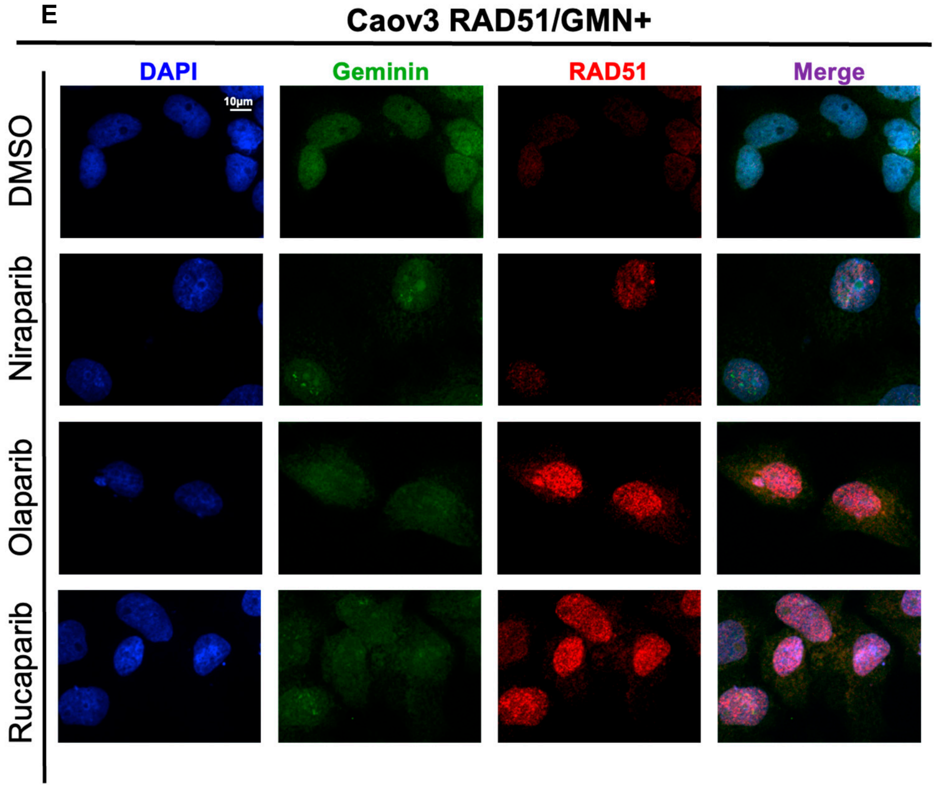
Caov3 RAD51/GMN+
DAPI
Geminin
RAD51
Merge
10μm
DMSO
Niraparib
Olaparib
Rucaparib

F

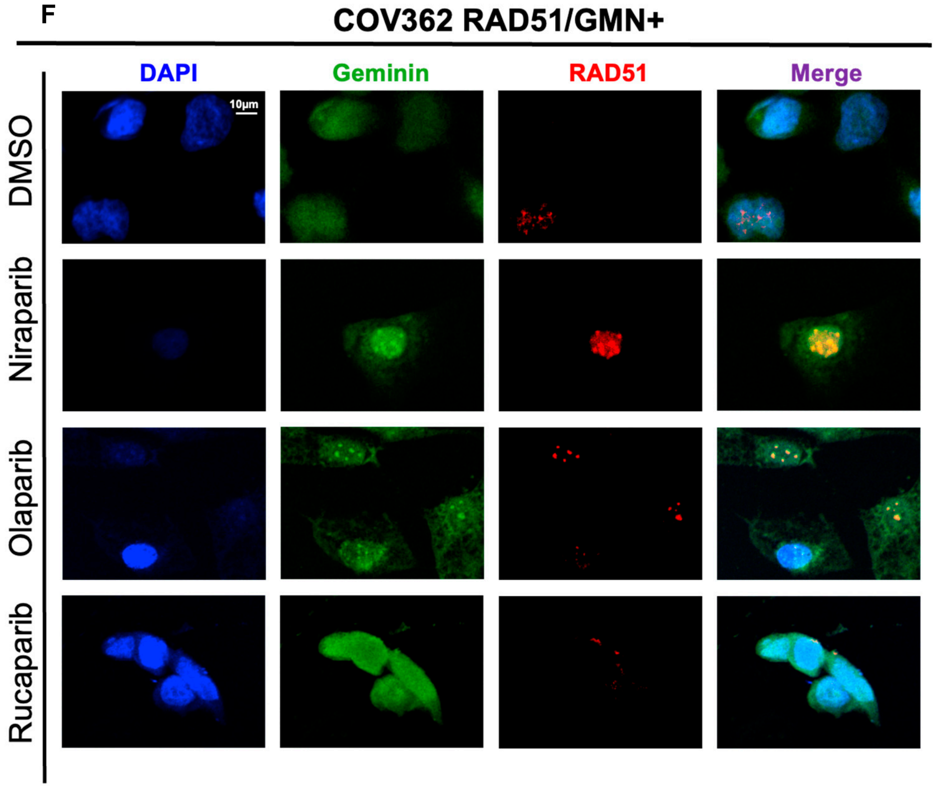
COV362 RAD51/GMN+
DAPI
Geminin
RAD51
Merge
10μm
DMSO
Niraparib
Olaparib
Rucaparib

Supplementary Figure S1. Determination of PARPi IC_{50} dose in HGSOC cells and assessment of DNA damage response. (A) Establishment of Niraparib, Olaparib, and Rucaparib IC_{50} dose following drug treatment for 72h in Caov3, COV362, and PEO1 cells using SRB assay (n ≥ 4 replicates per condition). (B) Table summarizing PARPi IC_{50} doses across HGSOC cells. Formation of γH2AX foci (C and D) and RAD51 foci in Geminin positive (GMN+) cells (E and F) were assessed using immunofluorescence microscopy following treatment with PARPi IC_{50} doses.

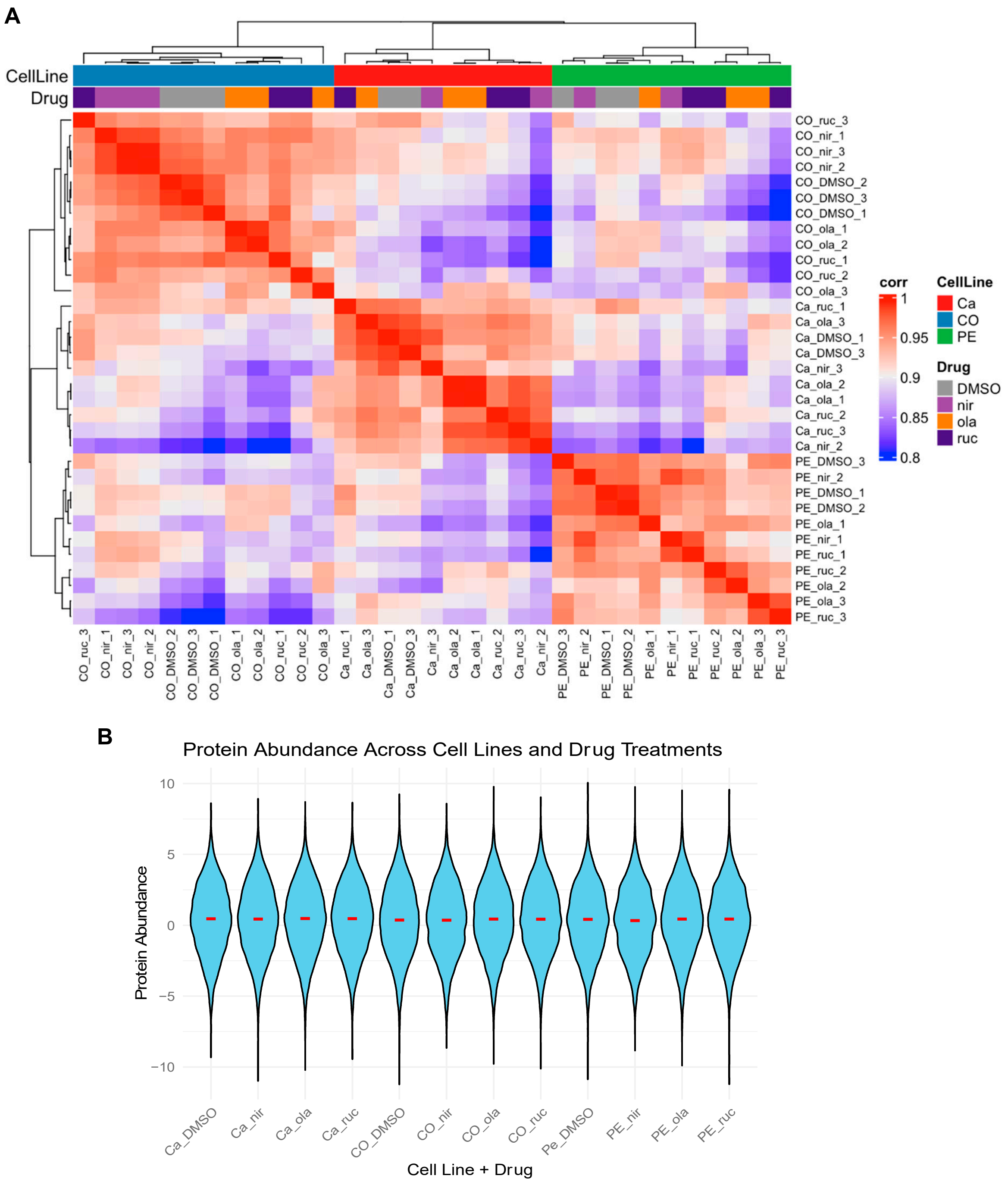

Supplementary Figure S2. Evaluation of sample reproducibility and data structure generated from mass spectrometry-based proteomics. Caov3 (Ca), COV362 (CO), and PEO1 (PE) cells treated with Niraparib (nir), Olaparib (ola), or Rucaparib (ruc) demonstrate high correlation across biological replicates (A). Distribution of normalized average protein abundances across samples (B).

A

Positive enrichment of pathways in COV362 vs Caov3

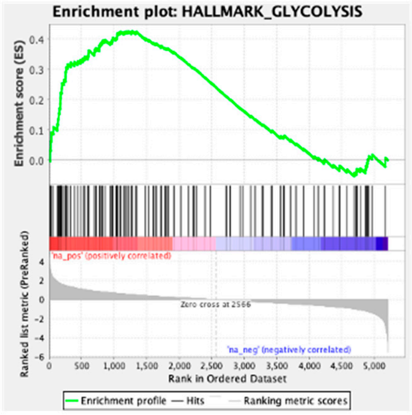

B

Negative enrichment of pathways in COV362 vs Caov3

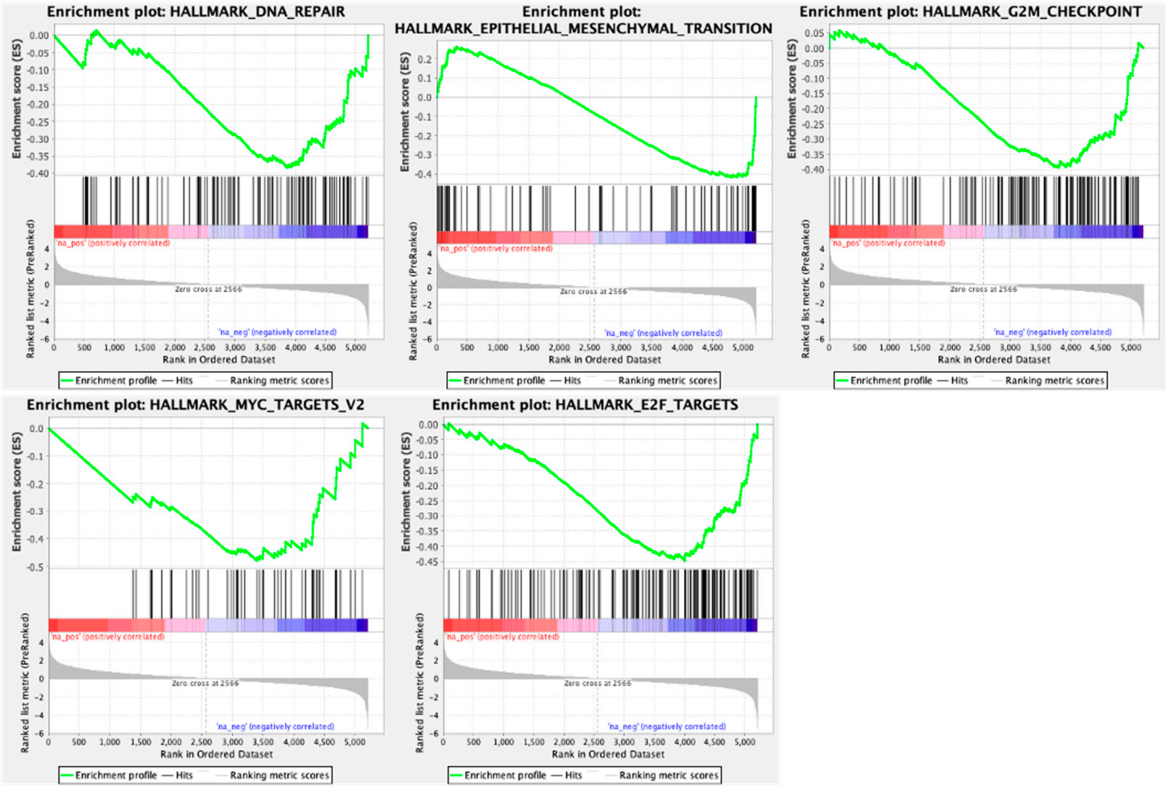

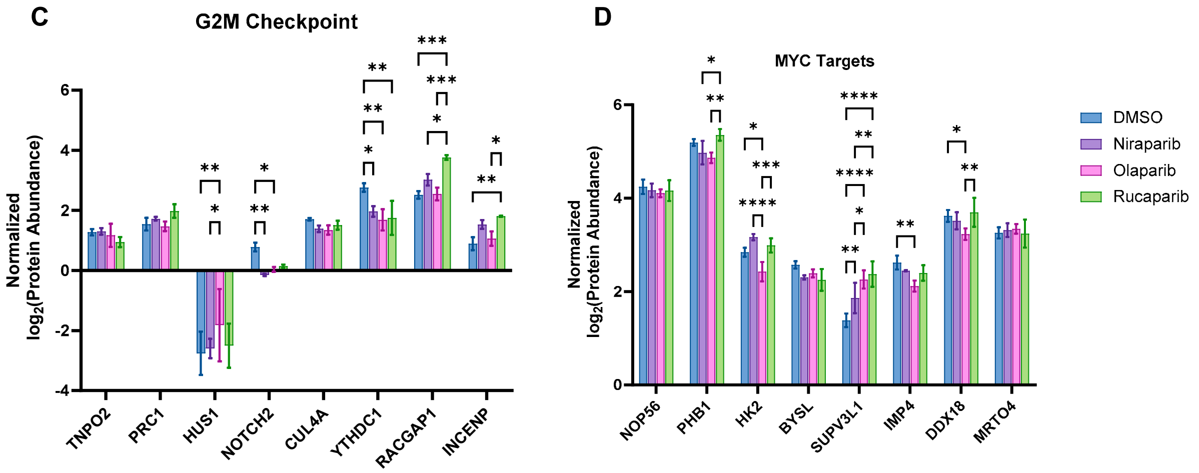

Supplementary Figure S3. Gene Set Enrichment Analysis (GSEA) identifies pathways enriched in COV362 cells vs. Caov3 cells. Positively (A) and negatively (B) enriched pathways in COV362 cells following GSEA analysis are shown. Perturbations to protein abundances following PARPi treatment are illustrated for G2M checkpoint (C) and MYC targets (D). Changes in protein abundances were assessed using two-way ANOVA followed by Tukey's multiple comparison test (* $p \leq 0.05$, ** $p \leq 0.01$, *** $p \leq 0.001$, **** $p \leq 0.0001$).

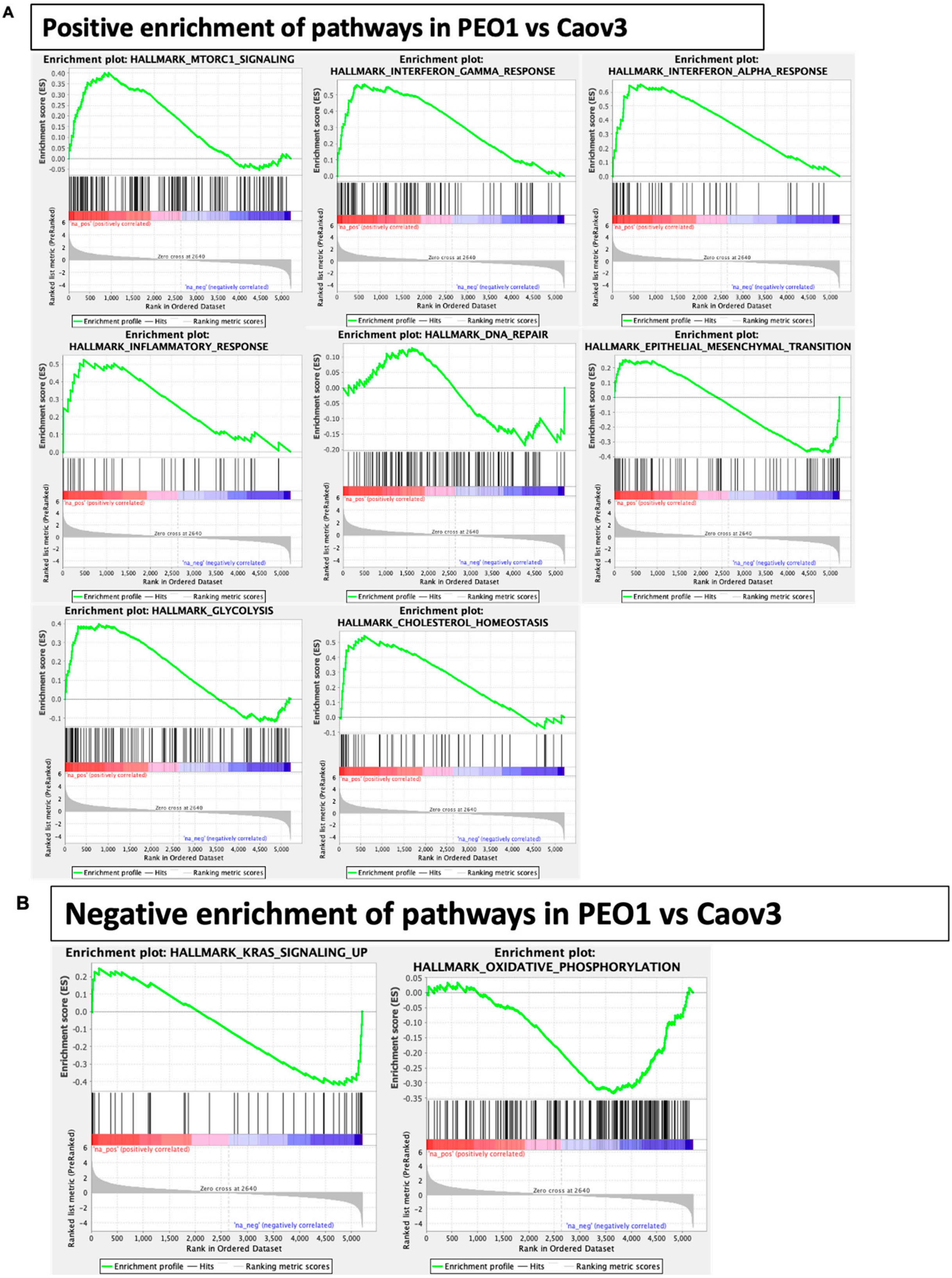
A
Positive enrichment of pathways in PEO1 vs Caov3
Enrichment plot: HALLMARK_MTORC1_SIGNALING
Enrichment plot: HALLMARK_INTERFERON_GAMMA_RESPONSE
Enrichment plot: HALLMARK_INTERFERON_ALPHA_RESPONSE
Enrichment plot: HALLMARK_INFLAMMATORY_RESPONSE
Enrichment plot: HALLMARK_DNA_REPAIR
Enrichment plot: HALLMARK_EPITHELIAL_MESENCHYMAL_TRANSITION
Enrichment plot: HALLMARK_GLYCOLYSIS
Enrichment plot: HALLMARK_CHOLESTEROL_HOMEOSTASIS
B
Negative enrichment of pathways in PEO1 vs Caov3
Enrichment plot: HALLMARK_KRAS_SIGNALING_UP
Enrichment plot: HALLMARK_OXIDATIVE_PHOSPHORYLATION
Enrichment score (ES)
Ranked list metric (PreRanked)
Rank in Ordered Dataset
'na_pos' (positively correlated)
'na_neg' (negatively correlated)
Zero cross at 2640
Enrichment profile
Hits
Ranking metric scores

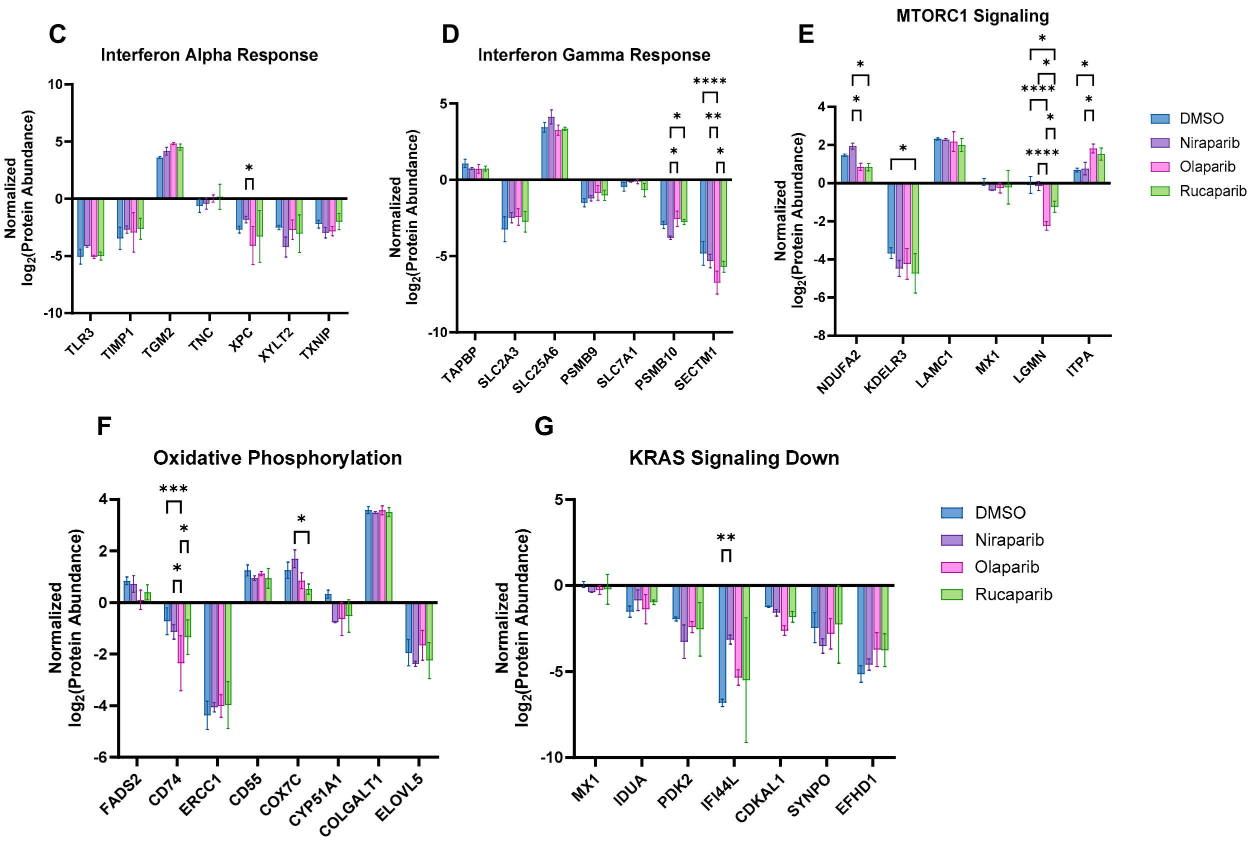

Supplementary Figure S4. GSEA identifies pathways enriched in PEO1 cells vs. Caov3 cells. Positively (A) and negatively (B) enriched pathways in PEO1 cells following GSEA analysis are shown. Perturbations to protein abundances following PARPi treatment are illustrated for distinctly enriched pathways (C-G) and assessed using two-way ANOVA followed by Tukey's multiple comparison test (* $p \leq 0.05$, ** $p \leq 0.01$, *** $p \leq 0.001$, **** $p \leq 0.0001$).

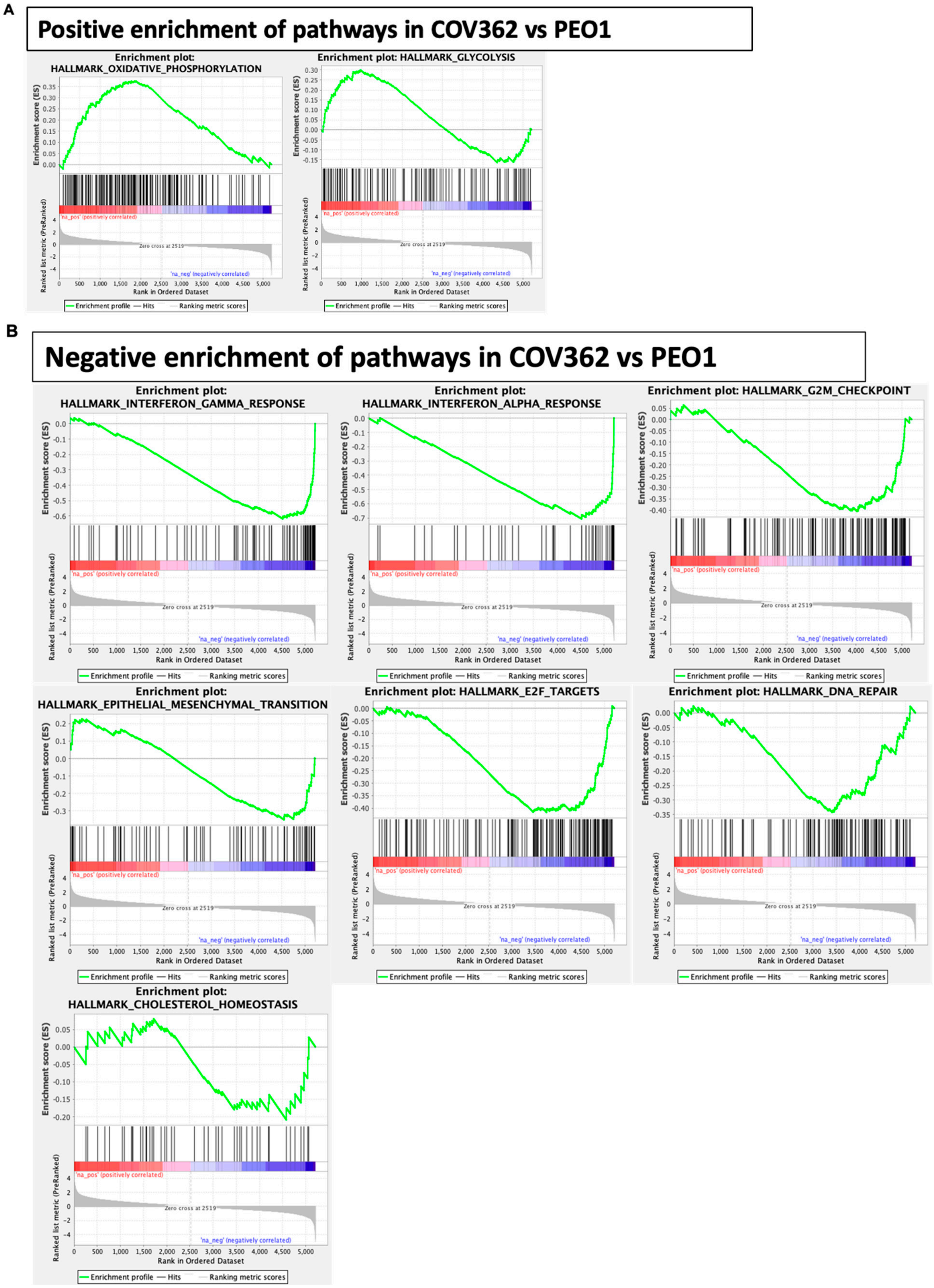
A
Positive enrichment of pathways in COV362 vs PEO1
Enrichment plot: HALLMARK_OXIDATIVE_PHOSPHORYLATION
Enrichment plot: HALLMARK_GLYCOLYSIS
B
Negative enrichment of pathways in COV362 vs PEO1
Enrichment plot: HALLMARK_INTERFERON_GAMMA_RESPONSE
Enrichment plot: HALLMARK_INTERFERON_ALPHA_RESPONSE
Enrichment plot: HALLMARK_G2M_CHECKPOINT
Enrichment plot: HALLMARK_EPITHELIAL_MESENCHYMAL_TRANSITION
Enrichment plot: HALLMARK_E2F_TARGETS
Enrichment plot: HALLMARK_DNA_REPAIR
Enrichment plot: HALLMARK_CHOLESTEROL_HOMEOSTASIS
Enrichment score (ES)
Ranked list metric (PreRanked)
Rank in Ordered Dataset
'na_pos' (positively correlated)
'na_neg' (negatively correlated)
Zero cross at 2519
Enrichment profile
Hits
Ranking metric scores

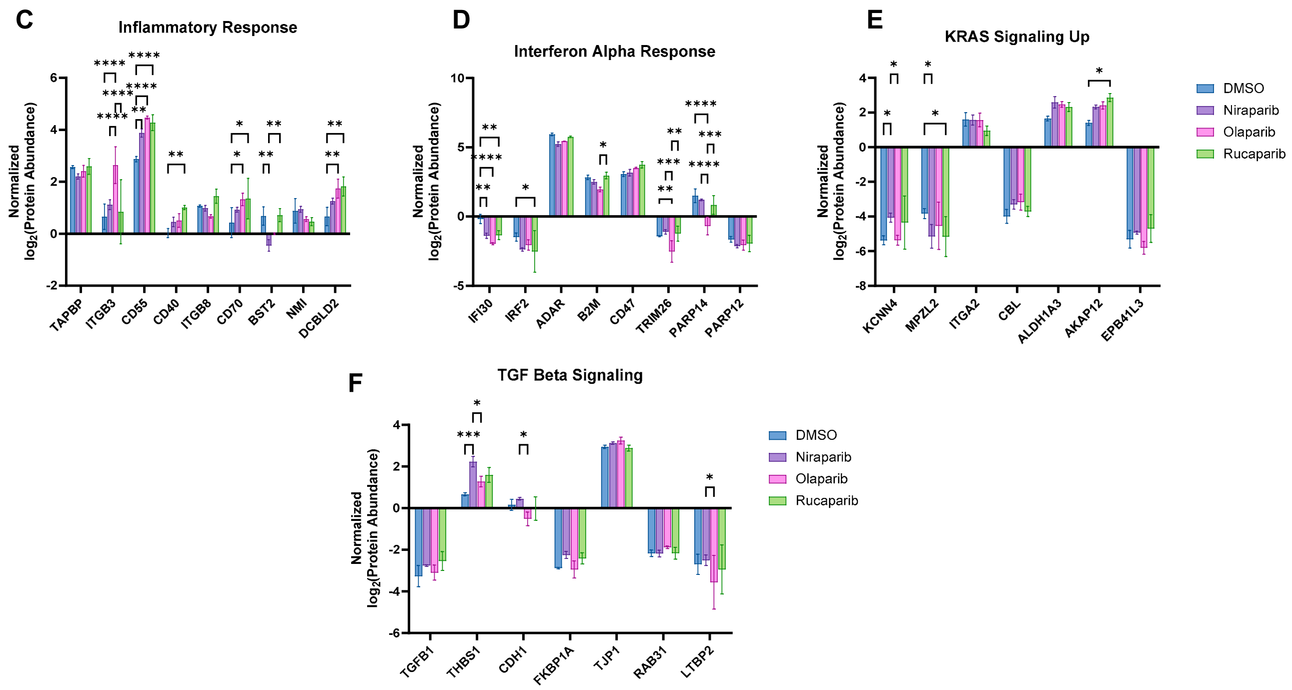

Supplementary Figure S5. GSEA identifies pathways enriched in COV362 cells vs. PEO1 cells. Positively (A) and negatively (B) enriched pathways in COV362 cells following GSEA analysis are shown. Perturbations to protein abundances following PARPi treatment are illustrated for distinctly enriched pathways (C-F) and assessed using two-way ANOVA followed by Tukey's multiple comparison test (* $p \leq 0.05$, ** $p \leq 0.01$, *** $p \leq 0.001$, **** $p \leq 0.0001$).

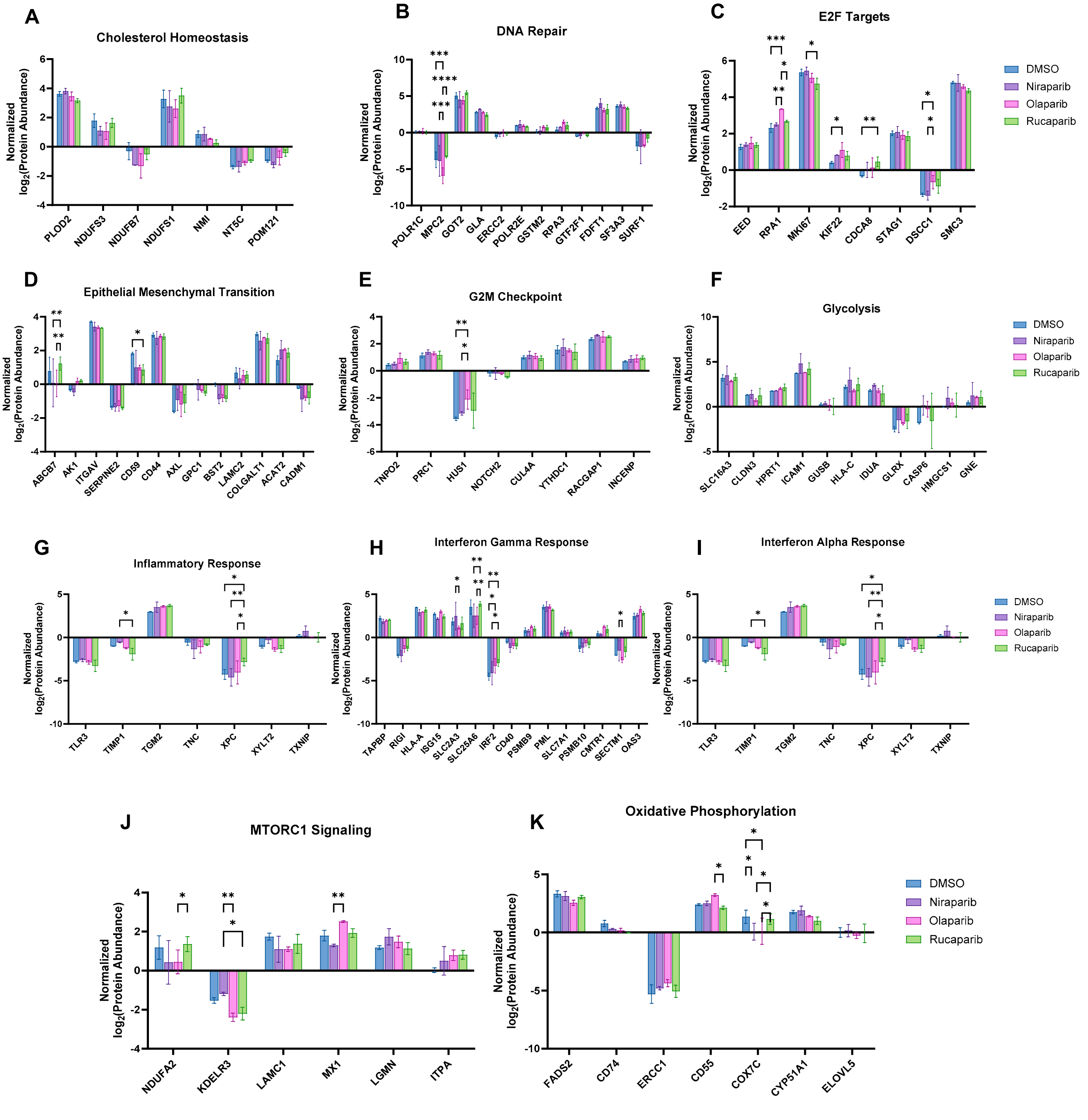

Supplementary Figure S6. Caov3 cells demonstrate minimal alterations to protein abundances following high-dose PARPi treatment. Top proteins belonging to enriched pathways identified from GSEA analysis in COV362 and PEO1 cells were assessed in Caov3 cells (A-K) using two-way ANOVA followed by Tukey's multiple comparison test (* $p \leq 0.05$, ** $p \leq 0.01$, *** $p \leq 0.001$, **** $p \leq 0.0001$).

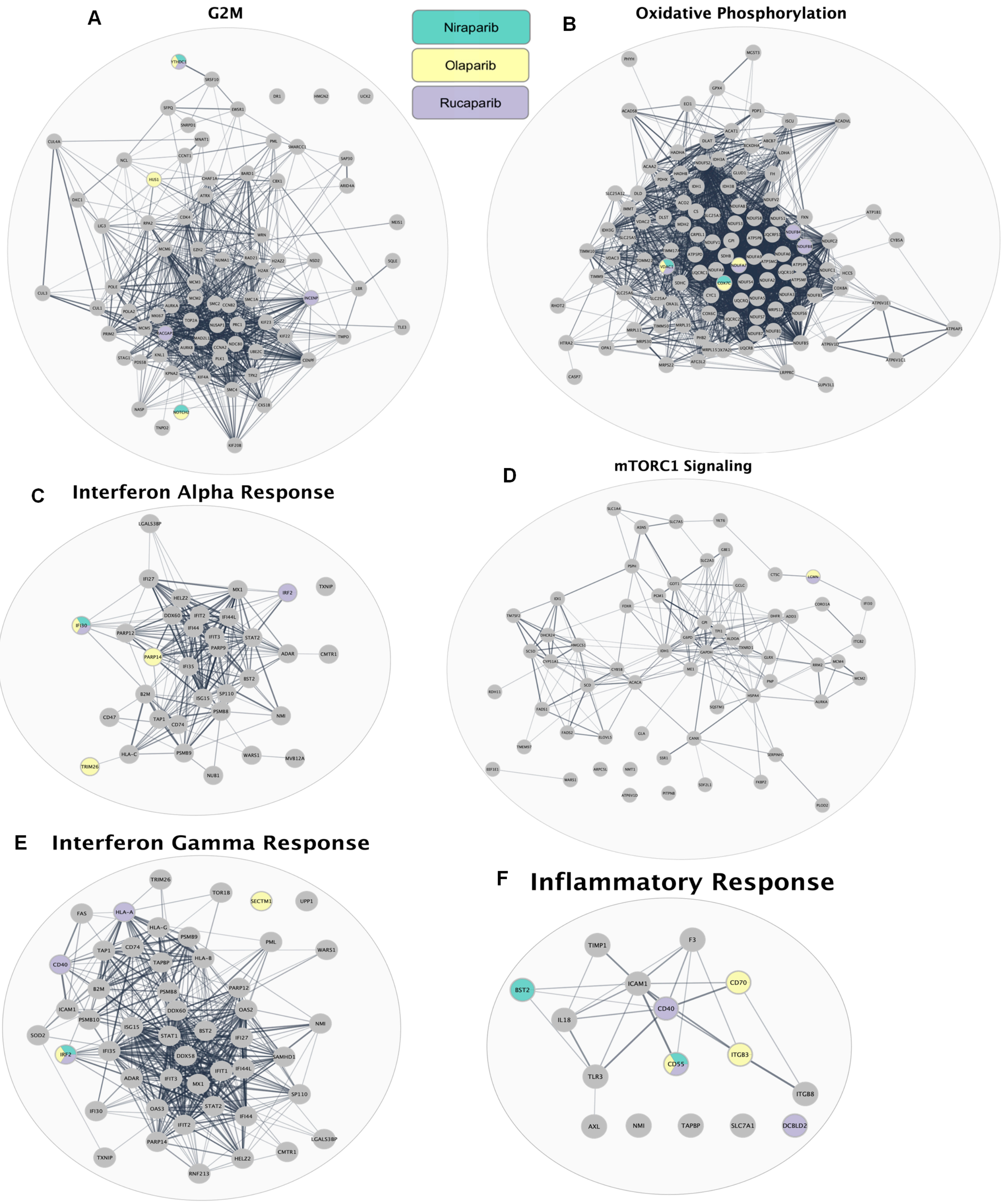
A
G2M
Niraparib
Olaparib
Rucaparib
B
Oxidative Phosphorylation
C
Interferon Alpha Response
LGALS3BP
IFI27
MX1
IRF2
TXNIP
HELZ2
DDX60
IFIT2
IFI44L
IFI30
PARP12
IFI44
IFIT3
STAT2
PARP9
ADAR
CMTR1
PARP14
IFI35
BST2
B2M
ISG15
SP110
PSMB8
NMI
CD47
TAP1
CD74
HLA-C
PSMB9
WARS1
MVB12A
TRIM26
NUB1
D
mTORC1 Signaling
E
Interferon Gamma Response
TRIM26
TOR1B
SECTM1
UPP1
FAS
HLA-A
HLA-G
PSMB9
PML
WARS1
TAP1
CD74
CD40
TAPBP
HLA-B
B2M
PARP12
PSMB8
ICAM1
DDX60
OAS2
PSMB10
NMI
ISG15
STAT1
BST2
SOD2
IFI27
IRF2
IFI35
SAMHD1
DDX58
IFIT1
IFI44L
ADAR
IFIT3
MX1
SP110
IFI30
OAS3
STAT2
IFI44
IFIT2
LGALS3BP
PARP14
CMTR1
TXNIP
HELZ2
RNF213
F
Inflammatory Response
TIMP1
F3
ICAM1
BST2
CD70
CD40
IL18
CD55
ITGB3
TLR3
ITGB8
AXL
NMI
TAPBP
SLC7A1
DCBLD2

Supplementary Figure S7. Protein-protein interaction networks of biological pathways identified through GSEA. Protein-protein interaction networks were developed using core enriched genes identified through GSEA analysis and STRING-db. Proteins significantly perturbed by PARPi treatment are highlighted (A-F).

Supplementary Tabel S1. TMT Label Scheme

TMT Label	Cells	PARPi	Set
129N	PEO1	DMSO	1
129C	PEO2	DMSO	2
130C	COV362	DMSO	1
126	COV362	DMSO	2
129C	COV362	DMSO	3
129C	Caov3	DMSO	3
130N	Caov3	DMSO	1
131	PEO1	DMSO	3
130N	PEO3	Niraparib	1
127N	COV362	Niraparib	3
127C	Caov3	Niraparib	2
128N	PEO1	Niraparib	2
128C	PEO1	Niraparib	3
130N	COV362	Niraparib	1
131	COV362	Niraparib	2
128N	Caov3	Niraparib	3
127C	Caov3	Niraparib	1
126	PEO1	Olaparib	3
127N	COV362	Olaparib	1
127C	COV362	Olaparib	3
127N	COV362	Olaparib	2
128C	Caov3	Olaparib	1
129N	Caov3	Olaparib	2
130C	PEO1	Olaparib	1
128N	PEO1	Olaparib	2
129N	Caov3	Olaparib	3
128N	COV362	Rucaparib	3
128C	Caov3	Rucaparib	3
129N	PEO1	Rucaparib	3
127C	COV362	Rucaparib	1
129C	Caov3	Rucaparib	1
130N	Caov3	Rucaparib	2
131	PEO1	Rucaparib	1
130C	COV362	Rucaparib	2
128C	PEO1	Rucaparib	2
